# Imaging of Hydrated and Living Cells in Transmission Electron Microscope: Summary, Challenges, and Perspectives

**DOI:** 10.1021/acsnano.5c00871

**Published:** 2025-03-29

**Authors:** Olga Kaczmarczyk, Daria Augustyniak, Andrzej Żak

**Affiliations:** † Institute of Advanced Materials, Wroclaw University of Science and Technology, 50-370 Wroclaw, Poland; ‡ Department of Pathogen Biology and Immunology, Faculty of Biological Sciences, University of Wroclaw, 51-148 Wroclaw, Poland; § Department of Material Science and Engineering, Massachusetts Institute of Science and Technology, Cambridge, Massachusetts 02139, United States

**Keywords:** live-cell, viability, bacteria, yeast cell, animal cell, radiolysis, radical scavenger, electron dose, liquid cell TEM, liquid phase TEM

## Abstract

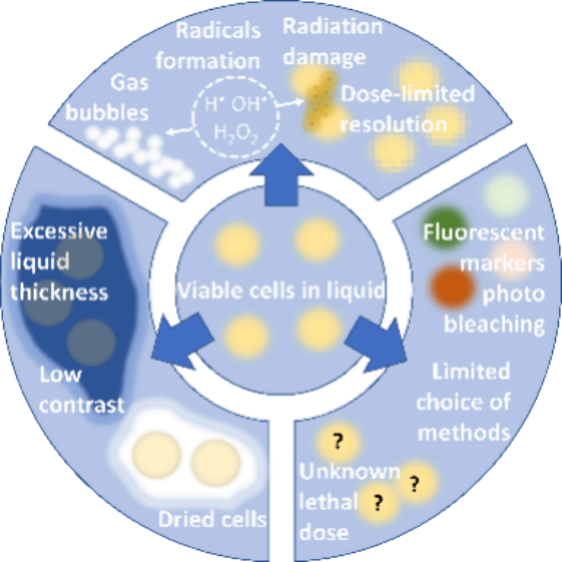

Transmission electron microscopy (TEM) is well-known for performing in situ studies in the nanoscale. Hence, scientists took this opportunity to explore the subtle processes occurring in living organisms. Nevertheless, such observations are complex—they require delicate samples kept in the liquid phase, low electron dose, and proper cell viability verification methods. Despite being highly demanding, so-called “live-cell” experiments have seen some degree of success. The presented review consists of an exhaustive literature review on reported “live-cell” studies and associated subjects, including liquid phase imaging, electron radiation interactions with liquids, and methods for cell viability testing. The challenges of modern, reliable research on living organisms are widely explained and discussed, and future perspectives for developing these techniques are presented.

## 1 Introduction

Light and electron microscopy are key methods for characterizing the structure of biological samples. However, due to the diffraction limit of light microscopy, it is not able to detect such delicate cellular structures as ribosomes (20–30 nm wide), actin filaments (7 nm), various intra- or extracellular vesicles (50–150 nm), and bacterial flagella (20 nm diameter) or pili/fimbriae (5–8 nm diameter).^1^ To image cell ultrastructure at near-nanoscale, researchers routinely use the method of imaging ultrathin sections of prefixed material.^2^ The most commonly used technique for such thin sections is transmission electron microscopy (TEM). Very similar results can also be obtained using scanning transmission electron microscopy (STEM), a technique more popular in materials science, which differs slightly in the way of generating images.

Due to the harmful effect of the electron beam on the delicate biological structures, samples are either chemically fixed, dehydrated, cut into sections, and then stained with heavy metal salts or cryogenically fixed, cut, and observed in a frozen form, using very restricted electron doses. Sometimes these methods are combined for better results.^3^ Nevertheless, these techniques share the requirement that the biological material must undergo some type of fixation, meaning it represents preserved rather than genuinely living tissue or cells. It must also maintain a thickness of no more than several hundred nanometers so the imaging resolution does not decrease due to high electron scattering on thick material. Imaging of ultrathin sections helps describe the detailed structure on a scale of single nanometers, even for volumetric analyses.^4^ Fluorescence and confocal microscopy are also crucial in cell studies at lower magnifications under varying environmental conditions. Specific structures or biological processes can be tracked by using a wide range of fluorescent dyes. Although, in particular cases, super-resolution techniques can achieve nanoscale resolution, in complex systems,^5^ they are not able to reach the resolving power offered by (S)TEM.^6^ This is one of the reasons that, for decades, researchers have been striving to image entire cells in an electron microscope.

This sometimes controversial approach of imaging living cells without any staining or fixation was called “live-cell”. The researchers continue to use this term, and so will we in this review. A different approach to imaging whole cells using (S)TEM is that of keeping the cells hydrated after fixation. This maintains the specific cell parts intact because of hydration (like cell membranes), and fixation makes them less sensitive to electron radiation at the same time. Nevertheless, the main drawback of imaging fixed hydrated samples is that it does not allow for in situ studies in the native state of the cell, limiting its use. The topic of TEM or STEM imaging of whole cells in a hydrated state started in 2008,^7^ a few years after the development of the modern, electrochemical silicon nitride-based liquid cell in 2003.^8^ Several reports in the literature focused on both hydrated^9−17^ and living cell studies,^7,18−40^ exploring topics such as general aspects of cell enclosure,^7,19,21,23,25,35,37,39,40^ radiation damage,^11,17^ contrast formation,^37,38^ and biological processes such as biomineralization,^20,26,28^ nanoparticle– and virus–cell interactions,^22,26,35^ antimicrobial mechanisms,^31−33^ or single-protein imaging.^10,17^ In the following years, different liquid cells and imaging techniques were utilized to study bacterial cells,^7,18−21,23,26,28,29,31,32,36,38−40^ animal cells,^22,30,35^ and yeast cells.^7,24,37^

**Figure 1:**
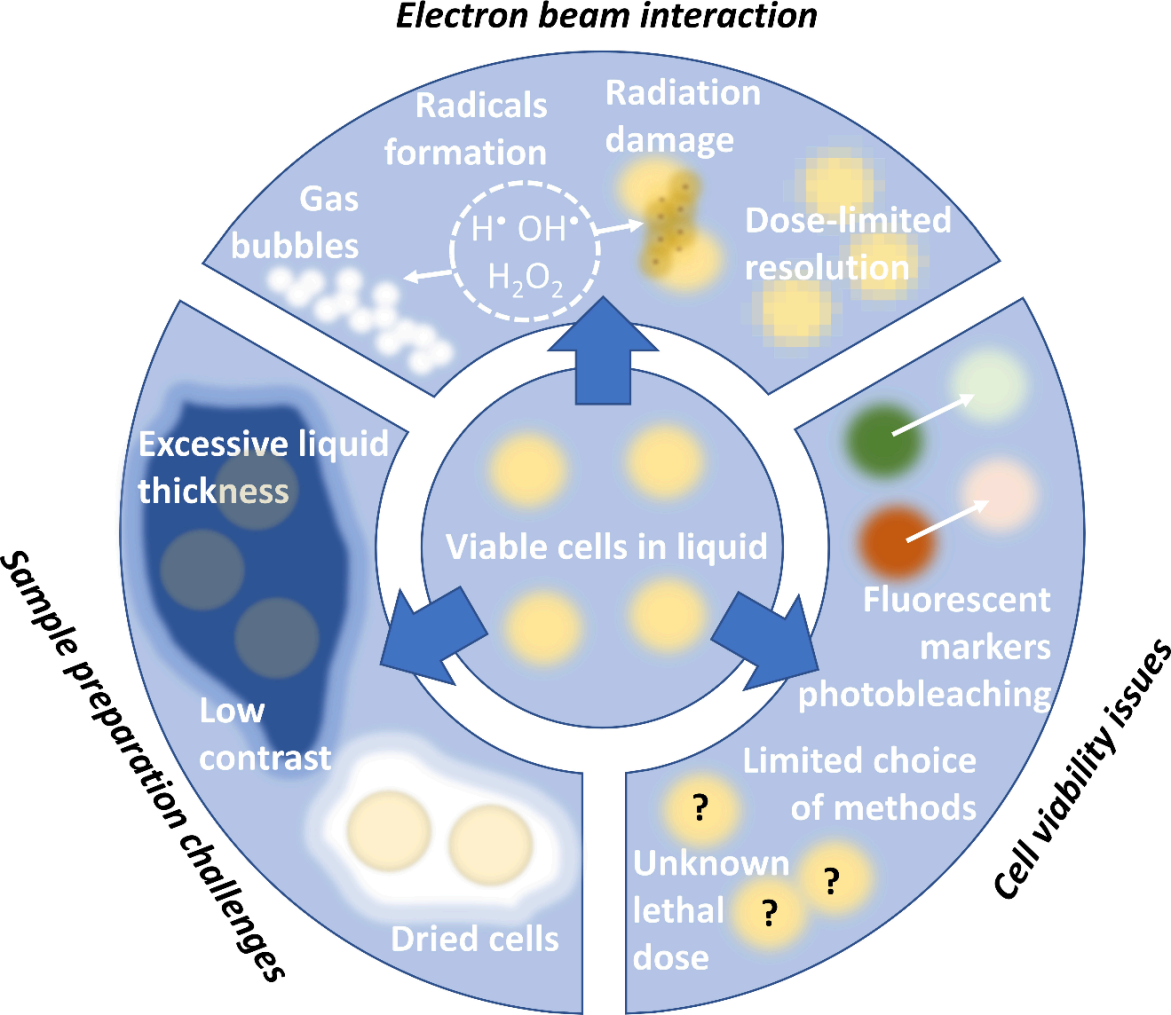
Diagram representing the main challenges of live-cell (S)TEM experiments. Once the viable cells in a liquid (presented in the center circle) are put in the microscope, a few problems must be faced to perform proper imaging. Three main challenges associated with the live-cell approach are electron beam interactions, cell viability issues, and sample preparation. The first one—electron beam interaction (upper part of the diagram)—responds to processes that occur in liquids upon ionizing electron irradiation: radiolysis followed by radical and bubble formation and sample damage. Because of that, low-dose imaging is required, which can significantly lower the resolution. The second challenge is complex sample preparation (left part of the diagram). The known methods are unreliable and do not provide perfect sample preparation. Because of that, either the liquid sample can have too much liquid (and therefore high thickness) or the liquid enclosure may fail and the cells would be subjected to drying. Generally, the cells covered with liquid give low contrast in (S)TEM. The last issue in live-cell imaging is cell viability during and after (S)TEM imaging (right part of the diagram). Commonly used fluorescence markers could undergo photobleaching or degradation, which may result in false viability interpretation. Additionally, many standard methods used for viability testing cannot be implemented on small (S)TEM samples; therefore, the choice of techniques is very limited. The last, but probably the most crucial, problem in live-cell imaging is the lack of systematic study of various species viability. Thus, the lethal electron dose is not specified.

The main controversy surrounding live-cell imaging is that an electron microscope’s environment is unsuitable for living cells due to internal vacuum and ionizing electron beam interactions with the sample. Therefore, this subject becomes complex, and a few challenges connected with sample preparation, electron beam interactions, and cell viability must be discussed (Figure 1). The first challenge involves sample preparation, specifically liquid cell enclosure of the cells. This technique has been explored by scientists for over 80 years now^41^ and is still being developed for various purposes,^42−45^ including live-cell imaging. After intensive improvement, this approach has become well-known, and commercialized devices can be easily used for nanomaterials, catalysis, or electrochemistry.^46^ However, in terms of live-cell conditions, things become more complicated. The issues connected to liquid cell preparation and viability testing include different effects of the used substrate (including, but not limited to, silicon nitride, graphene, carbon, and Formvar) on microorganisms, alterations in the efficiency of the liquid enclosure, the visibility of the area in both electron and fluorescence microscopes, and the low contrast of (S)TEM of relatively thick cells covered with liquid. Another significant challenge in live-cell imaging is the interaction between the electron beam and the specimen components. An electron beam as a highly energetic and ionizing radiation causes radiolysis processes in liquids,^47^ resulting in radicals and other reactive species formation, followed by internal cell damage and visible morphological changes.^7,18,21,38^ A critical trade-off exists between minimizing the electron dose to preserve cell viability and maintaining the resolution necessary for effective imaging. Furthermore, the feasibility of sustaining microbial life during TEM imaging remains an open question. The choice of viability assays suited to the TEM sample is very limited, and their reliability for the conducted experiments is still unsure. The lethal electron dose for living cells still needs specification regarding the imaging mode, cell types, and liquid cell substrate properties, as well as radiation damage, which needs a more careful explanation.

Upon the mentioned challenges, a few criteria need to be addressed for reliable live-cell imaging: (1) the cells need to be enclosed in a liquid cell and isolated from a high vacuum inside the microscope; (2) electron dose needs to be kept low to lower the unfavorable radiolysis effects and cell damage; (3) cell viability needs to be checked using known assays. All of these will be discussed in this review, beginning with a brief history and concepts of liquid cell (S)TEM imaging, radiation damage processes, and its prevention through the description of known live-cell literature, cell viability assays, and finally, conclusions with future perspectives.

## 2 Brief History and Basic Concepts of Liquid Cell (S)TEM

Many processes require a liquid environment for the process to occur. Notable examples include nanocrystal formation,^48^ growth of complex structures,^49^ catalysis,^50^ nanoparticle interactions^51−53^ and biological processes in living cells, which are the subject of this review. Nevertheless, liquid phase imaging may cause many problems,^42,44,47,54−56^ usually requiring complex sample preparation^57−59^ because an electron microscope operates in a vacuum, which can vaporize most standard liquids and solvents.

The very first reported attempt at liquid phase TEM imaging dates back to 1944,^41^ but the main modern techniques have been developed for the past 20 years. This method is now known under different, equivalent acronyms: liquid cell TEM (LC-TEM), liquid phase TEM (LP-TEM), and liquid environment TEM (LE-TEM).

Following this XXI century advancement, two main LP-TEM approaches can be distinguished as “open cell” and “closed cell”. The open cell approach involves imaging liquid samples without directly separating them from the vacuum environment. Such observations can be performed with the use of low-vapor-pressure ionic liquids,^60^ which remain in the liquid phase in a high vacuum in the microscope column. This method may be challenging to use in some cases as these specific solvents are not suitable for every experiment.^61^ Despite these limitations, ionic liquids were successfully implemented in inquisitive studies like (i) direct imaging in high-resolution of 1-butyl-3-methylimidazolium iodide (bmimI) ionic liquid on carbon nanotube substrate,^62^ (ii) in situ photocatalytic observations in *N*,*N*,*N*-trimethyl-*N*-propylammonium bis(trifluoromethanesulfonyl)imide,^63^ and (iii) gold nanoparticle growth in butyl-3-methylimidazolium bis(trifluoromethylsulfonyl)amide and trimethylpropylammonium bis(trifluoromethylsulfonyl)amide solution.^64^ However, ionic liquids cannot be utilized in live-cell observations as they exert a variety of harmful effects on cells, such as alteration of lipid distribution and cell membrane viscoelasticity, disruption of cytoplasmic, mitochondrial, and nuclear membranes, or even DNA fragmentation.^61^ Accordingly, the cells need an aqueous environment.

Another open cell technique is based on changing the environment inside TEM’s sample area. To achieve higher pressure in the specimen chamber, a differential pumping system is used.^65^ It makes observations on various liquids possible, but the pressure around the sample remains significantly lower than in the neutral conditions, which may inhibit certain processes, especially those occurring in living cells.^66^ Although the open-cell methods have been successfully used in multiple LP-TEM imaging studies, their applicability is restricted by the limited range of compatible solvents and the relatively high thickness, posing nanoscale and biological specimen observations.^42^

**Figure 2:**
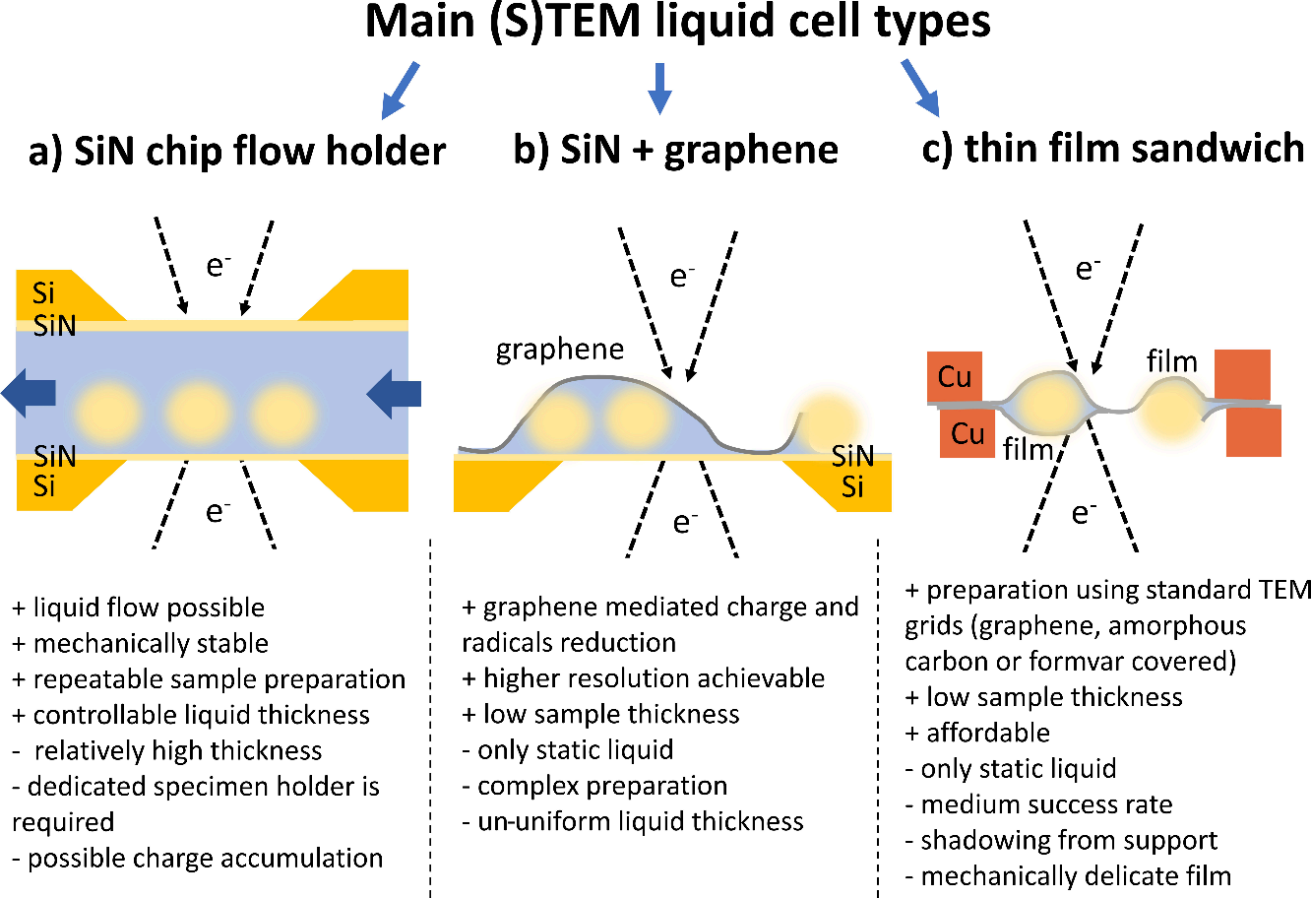
Graphical cross-sectional presentation of main liquid cell types used in live-cell research. (a) SiN-chip flow holder. The liquid flows between two stiff, electron transparent SiN membranes with a known spacer thickness. (b) Hybrid liquid cell made of SiN membranes and graphene sheet. The cells attached to the SiN window are covered with thin graphene. (c) Thin-film sandwich. Two standard TEM grids covered with graphene, amorphous carbon, or Formvar layer are sandwiched together to create liquid pockets between the thin films. The main advantages (+) and disadvantages (−) are listed under the schemes.

According to available knowledge, the only way to image whole cells in liquid is the “closed cell” approach, which is based on isolating the sample from a high vacuum by creating so-called liquid cells. Figure 2 shows a graphical presentation of the primary liquid cell types used in live-cell research, which are described in the following subsections.

The liquid cell strategy was used in several types of research. The first experiments were performed to observe the dynamic growth process of copper nanoclusters after electrodeposition.^8^ Further research expanded to the localization of biomolecules labeled with gold nanoparticles in liquids up to 10 μm thick using the STEM technique, which showed that exact observations of single molecules in whole cells are possible.^9^ Liquid cells were also used effectively to perform very precise imaging in the nanoscale, for example, the formation of nanoparticles from nanoclusters,^67^ ligand distribution on plasmonic nanoparticles,^53^ imaging complex polymer structures,^68^ and even 3D electron tomography of small colloidal assemblies.^52^ All types of liquid cells allow various in situ experiments, and the choice depends on the process characteristics. The following sections of this review will discuss its use in live-cell experiments. The reader can also find a detailed description of the LP-(S)TEM approach in distinguished reviews.^42−44,54,57,69,70^

### 2.1 SiN-Based Liquid Cells

One of the first materials used for liquid cells were silicon nitride (SiN) membranes.^8^ Modern SiN-based liquid cells usually consist of two separated SiN membranes (windows) of thickness ranging from 30 to 100 nm, which are supported by two silicon chips (Figure 2a).^9,71^ Prefabricated SiN liquid cells can either be glued, facilitating the liquid to remain static (commercially availabe K-kits),^7,52,72^ or assembled in a dedicated holder with a flow option.^46,73^ This method allows for using a wide range of liquids and has relatively convenient sample preparation, and observations can be performed in most typical microscopes. The technique is now commercialized with a few solutions available on the market.^46^ The SiN-based liquid cell type was the most widely used one for live-cell experiments because of its high mechanical resistance,^74^ the possibility for easy functionalization, and cell culture growth directly on the substrate.^19^ However, high-resolution images may be more challenging to achieve because of the high electron scattering, specimen thickness,^38,75^ and low electrical conductivity of silicon nitride.^76^ The main advantages and disadvantages of SiN liquid cells are listed in Figure 2a.

### 2.2 Graphene Liquid Cells (GLCs)

Despite the aforementioned SiN membranes, graphene can also separate liquids from the vacuum. The first GLC created in 2012 was used for observations of platinum nanocrystal growth.^48^ In this case, the liquid is enclosed between two graphene sheets forming GLC. This material, compared to SiN, is characterized by remarkable electric conductivity, high flexibility, and low atomic number.^70,77−81^ Owing to these properties, graphene provides higher spatial resolution and reduces charge accumulation.^82−84^ In addition, graphene and its derivatives have been shown to act as efficient radical scavengers, helping to minimize radiation damage to sensitive specimens.^84^ Furthermore, the chemically reactive surface of graphene enables its tight adhesion to both prokaryotic and eukaryotic cells.^85^ The ideal GLC could be made by even using a single graphene layer. However, sample preparation remains challenging. Multilayer sheets of the material are easier to handle, prevent eventual leakage of liquid caused by defects, and do not disturb observation.^11^ The average thickness of a multilayer graphene sheet is about 1 nm, which is much more favorable than the 30–100 nm thick SiN window.^63^

The GLC procedure is quite complex and can be accomplished using two general approaches: (i) by sandwiching two graphene-coated grids with liquid between them or (ii) by covering the sample on a graphene substrate with free-floating graphene.^86−88^ In the first method requiring the use of two graphene-coated grids, the grids are pressed and van der Waals forces between the graphene layers facilitate the formation of liquid pockets. Currently, GLC’s fabrication with the use of two graphene-coated TEM grids appears to be one of the most popular techniques used.^28,48,87,89−91^ In the second approach, the graphene sheet is transferred on the first grid to cover the sample. Transfer of graphene is probably the most complicated part of GLC fabrication, prompting ongoing research into improved methodologies.^57^ For example, free-floating graphene can be collected by the sample from below^17^ or by lowering the liquid level.^40,92^ Graphene can also be placed on the sample on a drop of liquid using a metal or nylon loop^88,93,94^ As described above, the main disadvantages of using graphene for LP-TEM imaging are complex sample preparation and un-uniform thickness. Figure 2c illustrates a visual representation of the GLC sandwich, highlighting its key advantages and disadvantages.

### 2.3 Other Liquid Cell Types

Another liquid cell type used in live-cell research is a combination of the methods mentioned above in which both SiN membrane and graphene are used.^17^ The membrane is supported by a silicon microchip from the bottom, and the liquid is covered with graphene from the top. This combination has advantages as the cells are placed on stiff SiN and graphene can reduce charging effects and unfavorable radiolysis products. No unique sample holder is required in this case, but the preparation remains complex.^57^Figure 2b shows a graphic showing this type of liquid cell with the main advantages and disadvantages listed.

It is worth mentioning that a less sophisticated “sandwich” method is also effective for liquid enclosure between two Formvar^35,95^ or carbon films.^31,32,36,96−98^ This technique represents the simplest and most affordable way to prepare LP-TEM samples; however, it is hindered by a lack of uniform thickness, worse film adhesion, and lower resistance to mechanical damage. Figure 2c shows a graphical presentation of a liquid cell prepared using the sandwiching method with the main advantages and disadvantages listed.

### 2.4 Sample Preparation for Live-Cell and Hydrated-State Observations

All of the liquid cell materials mentioned above can be used for live-cell experiments or observations in the hydrated state. The cells could be confined in liquid by simply sandwiching two graphene-, carbon-, or Formvar-coated grids,^28,31,32,35,36^ collecting free-floating graphene,^17,30,40^ or between SiO_2_ or SiN membranes.^7,19,21,24,33,99^ In the case of imaging fixed, hydrated cells, the sample requires a specific treatment based on well-established protocols^2^ before liquid cell enclosure. Nevertheless, some notable modifications in sample preparation for both live-cell and hydrated-state observations are worth mentioning.

**Figure 3:**
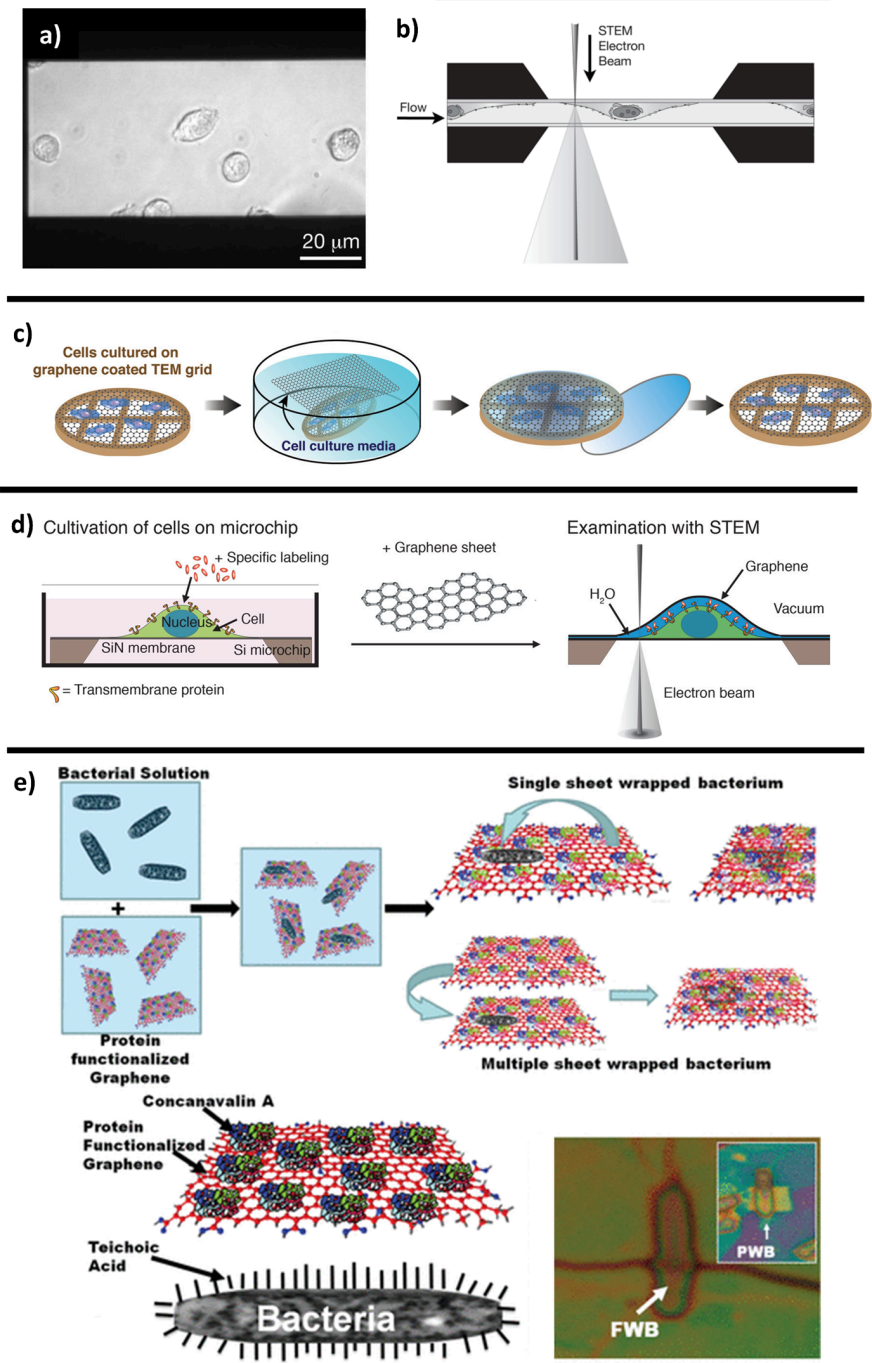
Graphical presentation of sample preparation methods dedicated to cell observations in LC-(S)TEM. (a) Cells attached to a positively charged poly-l-lysine layer on the upper SiN window of the liquid microwell. The liquid flow does not disrupt the adhered cells. (b) Light microscopy image of COS7 cells (African green monkey kidney fibroblast-like cells) on SiN window after 5 min incubation. Adapted with permission from ref (99). Copyright 2011 The Authors, published by Royal Microscopical Society. (c) Cell culture on Si microchip with electron-transparent SiN membrane. When the cells grow to the desired density, they can be covered with graphene for LC-STEM imaging. Adapted with permission from ref (17). Copyright 2017 American Chemical Society. (d) GLC preparation of cells grown on a graphene-coated TEM grid. Liquid enclosure is achieved by collecting the free-floating graphene from the cell culture medium solution. Once the graphene covers the cells with solution, the filter paper blots the excess liquid to ensure a sufficient enclosure between the sheets. Adapted from with permission ref (11). Copyright 2015 American Chemical Society. (e) Liquid cell of bacteria using protein-functionalized graphene (middle left image). ConA protein shows a specific affinity to the teichoic acids, which are cell membrane components (bottom left image). First, the functionalized graphene is mixed with bacteria in a solution, where graphene attaches to the cells thanks to interactions between the ConA protein and the cell wall. The process continues until the graphene wraps the cells with either a single layer (upper right image) or multiple layers (middle right image). The wrapping efficiency can be checked using a light microscope as shown in the inset photo (bottom right image, FWB – fully wrapped bacterium, PWB – partially wrapped bacterium). Adapted with permission from ref (18). Copyright 2011 American Chemical Society.

In the case of often used SiN-based liquid cell holders, it is recommended to coat one of the chips with a thin layer of poly-l-lysine or (3-aminopropyl)triethoxysilane (APTES).^13−16,20,23,24,26,27,37,99,100^ The resulting layer increases hydrophilicity and provides a positively charged surface, which promotes cell attachment (Figure 3a) by electrostatic interactions with negatively charged cell membranes.^101^ Additionally, commercially available biofunctionalized chips featuring positively charged SiN membranes offer a ready-to-use alternative.^20^ Preprepared SiN membranes are also a suitable substrate material for cell growth (Figure 3b).^17^ The cultured cells can then be enclosed with a solution using graphene (Figure 3c). This approach allows the (S)TEM experiment to start once the cells achieve the desired density. Another improvement for SiN-based liquid cells was the development of multiwindow devices for better sample imaging,^39^ where the imaging area is increased and divided into smaller regions by grids. Grid bars function as focusing aids that help get a proper focus without the risk of creating artifacts and decrease the bulging effect.^75^

The cells can also be grown efficiently on a graphene-covered TEM grid. This particular approach was shown in a work from 2015, which focused on the development of a GLC technique for observations of hydrated H3N2 influenza viruses and Madin Darby canine kidney (MDCK) cells.^11^ Additional fluorescence imaging using GFP proved the cell’s living activity on a graphene-covered holey carbon TEM grid. Once the cells were grown on the substrate, GLC could be formed by collecting floating graphene directly from the cell culture medium solution (Figure 3d). In this case, before GLC fabrication, cells were extracted and fixed on the grid but remained hydrated after being covered with a multilayer graphene sheet.

Another interesting approach in preparation for LC-TEM is the usage of protein-functionalized graphene.^18^ Graphene oxide is rich in carboxyl groups (−COOH), so it can be covalently bonded with amino groups from proteins^102^ and, also, those that can bind to the outer components of the cell. An example of such a protein used to enhance bacteria enclosure in graphene is Concanavalin-A (ConA), which shows a specific affinity for the bacterial cell wall.^103,104^ This allows the cells to be wrapped in graphene, as shown in Figure 3e. The graphene-covered cells can then be immobilized on SiN membranes to perform (S)TEM imaging in a hydrated state. Multilayered graphene enclosures provide additional leakage prevention and also provide radical scavenging properties.

To summarize, all liquid cell types have unique properties and have been used for cell imaging. Selecting the best liquid cell type for live-cell or hydrated-state experiments is difficult, as the choice depends on experiment specifications. SiN-based liquid cells in a dedicated holder are (for now) the only option for experiments that require flow or liquid exchange (for example, from water to organic solution or nanomaterial dispersion). Even though the dielectric membranes enhance the charge accumulation and increase electron scattering, they can provide a higher liquid thickness (up to 10 μm^9,105^), which is crucial for micrometer-sized cells. Additionally, medium could flow through the holder without disturbing the cells attached to poly(l-lysine) or APTES layer. There are some reports on the negative impact of poly(l-lysine) on bacteria,^106,107^ so it could be considered to choose other ways to provide their adhesion to SiN membranes. The holder and microchips are relatively expensive compared to other liquid cell techniques but provide unparalleled possibilities and sample preparation reproducibility. In the case of so-called K-kits, it is crucial to choose a proper spacing distance between the SiN windows.^72^ Micrometer-sized cells will not be able to go through nanometer-sized spacing, and even for 2 or 5 μm spacing there is a risk of blocking the entry by a few cells stuck together. Therefore, SiN-based liquid cell holders seem to give the most universal opportunities for cell imaging, of course, with the restriction of dose and thickness limited resolution.^44^ Additional use of the radical scavengers^108−110^ and future development of additional conductive coatings^68,84^ may even increase the possibilities.

From the historical point of view, most experiments on cells (either living or fixed) were performed using SiN-based liquid cells.^13,14,16,20,21,23,24,26,27,37,99,100^ However, over time, the use of graphene has increased due to its low scattering factor and scavenging properties, which enable higher-resolution imaging and help prevent cell damage.^11,58,84,88,111,112^ Use of GLCs is therefore highly recommended for sensitive living cell imaging and nanoscale; high-resolution research, for example, cell-nanomaterials interactions and single molecule analysis;^17^ or materials nucleation and growth within a cell.^28^ When liquid flow is not necessary, GLCs or their hybrid version with a SiN bottom substrate should be considered a method of choice, especially for live-cell experiments. GLCs’ main drawback is that the sample preparation is more complex and maintains low reproducibility.

The most affordable and convenient carbon and Formvar film sandwiches^31,32,35,36^ can also be considered a good alternative to SiN and GLCs, especially for preliminary studies of cells, where high resolution or viability are not considered. It is a convenient method for checking the overall morphology and selecting the cell density for the experiment. Thin film sandwiches can also help evaluate the solution’s reaction to the electron beam. For example, it can help determine whether specific solution components will crystallize upon electron irradiation. However, for a more detailed analysis where unfavorable radiolysis effects play a crucial role^47^ it is more recommended to use graphene sandwiches instead.

## 3 Electron Beam Interactions in Liquid Phase Imaging

The interaction of the electron beam with the sample is the absolute basis for high-resolution imaging. While elastic scattering preserves the energy of the original electrons and only changes the direction of electron motion (which is why it does not transfer any significant energy to the atoms), inelastic scattering is responsible for practically all of the so-called “beam damage”, i.e., the unfavorable interaction of the electron beam with the original structure of our sample. Although both scattering mechanisms are quite well understood, their mutual influence on the image is still being analyzed.^113^ However, the most crucial inelastic scattering is related to three main mechanisms of sample damage:^114^

(i) knock-on damage, i.e., moving atoms under the influence of a beam, causing point defects, or even knocking the atom out of its original position (it can then be sputtered);
(ii) sample heating, which is usually negligible, but in the case of materials that do not conduct heat and electric charge well or are extremely sensitive to even a minimal increase in temperature, this factor should also be considered;
(iii) radiolysis, i.e., ionization or breaking of chemical bonds.

### 3.1 Radiolysis in Liquid Cell (S)TEM

While the most closely monitored and considered issue in engineering and materials sciences is usually knock-on damage, special attention should be paid to radiolysis phenomena in the case of imaging soft materials and biological specimens. It is imperative in liquid cell electron microscopy,^54,115^ which is necessary for cell liquid enclosure. Although a wide range of solvents and solutions can be used in LC-TEM,^116^ when imaging hydrated biospecimens, the typical sample environment will be water and popular buffer solutions. Fortunately, among many potential environments, water has been best characterized as the subject of liquid cell analyses. It has been shown that depending on the electron dose used, the pH changes quite significantly, and the various water radiolysis products are formed.^45,47,117^ The effect of local illumination of the sample with an electron beam leads to the formation of a significant gradient in the chemical composition of the solution, which cannot be ignored.^115^ Among the products of water radiolysis, it is worth mentioning, among others, H^•^, OH^•^, and H_2_O_2_.^117,118^ With higher doses of electrons, the formation of H_2_ bubbles can even be observed, which are sometimes used to test the presence of liquid inside the tested cell.^55^ This gas bubble type has also been used as a late and final indicator of cell death.^119^

There is no doubt that controlling and tracking the electron dose within a specimen is essential for understanding and limiting beam-induced damage processes.^120^ This type of issue was raised in the early years of electron microscopy development by Laszlo Marton, who noticed the shrinkage of a biological section under the influence of the beam.^121^ The topic was regularly discussed in the community but had the most significant impact on the field of biological microscopy.^122^ It was crucial for the development of cryogenic electron microscopy (cryoEM) and led to specific guidelines for the doses at which single biomacromolecules can be imaged without significantly affecting their structure.^123^ With the development of technology, new methods for measuring beam current and electron dose have appeared,^124−126^ ultimately leading to the development of commercial solutions capable of tracking the cumulative dose throughout the entire experiment.^127^

Beyond conventional TEM microscopy, some researchers preferred to perform liquid phase experiments using the STEM mode.^9,10,17,30,128^ This approach allows for a slightly different method of controlling the electron dose on the sample. Instead of dividing the total amount of electrons (and therefore the beam current) by the illuminated area,^129^ it is enough to define the scanning beam current, dwell time (the time the beam remains at each scanning point), and the size of the pixel and the probe itself.^124^ Additionally, this technique allows for local impact on the sample, restricting electron exposure exclusively to the site under observation. However, there is no doubt that radiolysis products formed at one place of the sample can interact within a range of even micrometers from the beam.^45^ This implies that despite the advantages of the STEM mode, such as localized dose delivery, higher contrast, and the possibility of imaging micrometers-thick samples, it does not consistently surpass conventional TEM.^130^ TEM, in certain scenarios, provides benefits such as faster image acquisition and more uniform illumination.^131^ It is often pointed out that it is necessary to precisely calibrate your microscope because factory calibration errors can reach several times.^56^ Usually, the key to precision is to measure the beam current using a physical Faraday cup placed at the sample location.^129,132^ It happens that some manufacturers place this type of accessory directly in the sample holder.^124^

An issue inextricably related to electron dose is the associated resolution limitation, which affects both the TEM and STEM modes.^56^ The matter is particularly complex for samples of considerable thickness. Egerton determined the voxel dose-limited resolution of a sample containing regions differing in density by only 10%, for a sample of 1 μm thickness at a resolution of about 17 nm and for a sample of 5 μm thickness at only 70 nm.^133^ While these values may appear unfavorable compared to those associated with high-resolution electron microscopy, this discrepancy arises from the specific conditions of our analysis. Accordingly, this results from the fact that the case analyzed by us and the author assumes minimal differences in density in a sample consisting of light elements and popular high-resolution imaging is usually carried out on periodic, crystalline structures with higher atomic number and density.

It is also worth remembering the different mechanisms of image formation in TEM and STEM, and consequently, a different mechanism of delivering electrons to the sample. In TEM, the given dose rate covers the field of view and a wider area of the sample (illuminated by the electron beam). Without precise mapping of the beam area,^127^ it is easy to interact with electrons in slightly further areas of the sample, but the electrons are delivered continuously and simultaneously. In the STEM method, the narrow, convergent scanning beam stops on subsequent fragments of the sample for a specified dwell time, and for each scan, each of the pixels of the image receives only one packet of electrons.^124,131^ In this case, the nature of the energy supply is impulsive and not continuous. It is, therefore, difficult to directly compare both mechanisms of delivering the electron dose, and with an identical dose rate, one cannot expect the exact behavior of the mechanisms of interaction between the beam and the material.^47^ However, the undoubted advantage of STEM methods is that the scanning beam only minimally extends beyond the frame area, facilitating separate observations from the minimum dose in different sample parts. In addition to easier control of the locally delivered, albeit pulsed, dose of electrons, the advantages of STEM methods include the possibility of broad control of the obtained contrast by using variable camera lengths or the possibility of obtaining phase contrast using multisegment detectors and the integrated differential phase contrast (iDPC) technique,^134^ as well as the growing possibilities of 4DSTEM phytography imaging.^135^

### 3.2 Mitigating the Radiolysis Impact on the Sample

**Figure 4:**
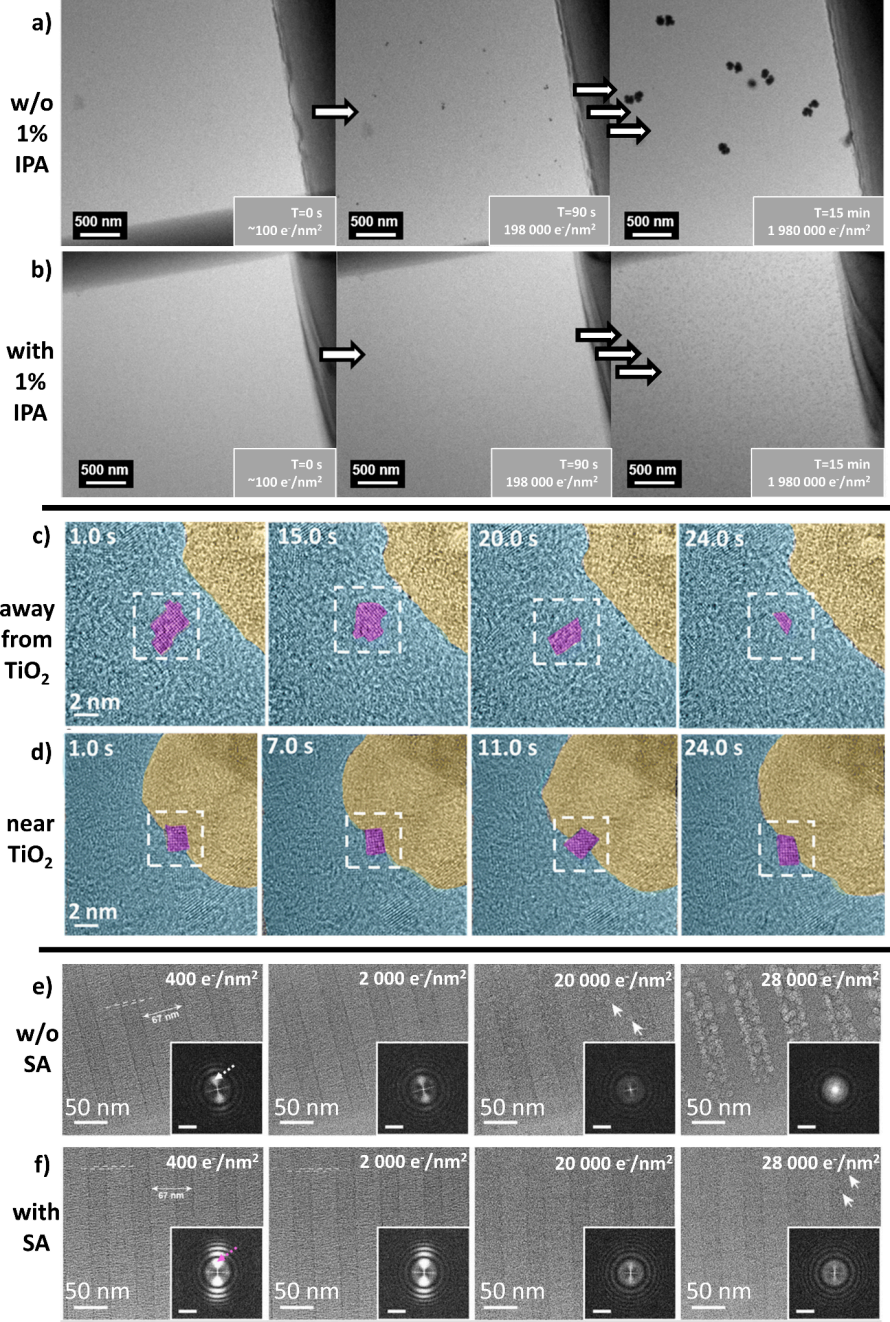
Examples of visual differences using radiolysis scavengers in LC-TEM and cryoEM: (a, b) 2.4 kDa polyethylene glycol (PEG) imaged in the absence (a) and presence (b) of 1% IPA under dose up to 1980000 e^–^/nm^2^, showing growth of radiolysis-driven particles in the absence of IPA (a). Adapted with permission from ref (109). Copyright 2021 American Chemical Society. (c, d) Ice-crystal (false-colored to violet) dissolution sequences on and off the TiO_2_ (yellow)–water (blue) nanointerface in GLC, as observed after electron-beam exposure at cryogenic temperatures, away from the TiO_2_ nanoparticle interface (c) and at the TiO_2_ nanoparticle surface (d), confirming the gradual reduction in ice crystal size away from TiO_2_ (c) and ice crystal’s stability near TiO_2_ under the electron beam (d). Adapted with permission from ref (139). Copyright 2023 Royal Chemical Society. (e, f) Cryo-TEM images of collagen fibrils without additive (e) and with the addition of 0.1 M SA (e) after 400, 2000, 20000, and 28000 e^–^/nm^2^ of electron exposure. Dashed lines indicate the ∼1.5 nm thick filaments, and white arrows highlight the appearance of gas bubbles. The insets are corresponding FFT images; scale bar = 0.5 nm^–1^. Adapted f with permissionrom ref (141). Copyright 2024 American Chemical Society.

While the effect of an electron beam on a static liquid sample is well characterized, the situation is much less clear when the medium flows around the sample, which is available in numerous LC-TEM methods. It could be assumed that the flow of the medium around the imaged area (e.g., bacteria) will significantly reduce the concentration of harmful radiolysis processes of water and other media; however, due to the speed of radical generation, not all of them could be effectively removed.^136^ On the other hand, the liquid flow will not improve the situation inside closed spaces, including imaged cells. There is no doubt that the topic of the flow of the medium at the maximum lethal dose of electrons requires not only modeling^136^ but also further experimental studies. However, an alternative method to reduce harmful beam-induced radiolysis involves the use of additional substances, called scavengers, which absorb harmful reactive species and reduce their detrimental effects on the sample. For the first time, such an effect was observed for one of the materials from which a liquid cell can be made—graphene.^84^ Some early research on GLC has shown that this sample exhibits about 10 times greater resistance to electron dose than the cryoEM sample, potentially more advantageous due to the lower temperature.^111^ Some works have even indicated the prospects of GLC for live-cell imaging.^137^ A similar approach was used to image the ultrastructure of amyloid fibrils, which confirmed the high prospects for using GLC for dose-sensitive samples.^112^ Similarly promising is using liquid scavengers, which slightly change the chemistry and radiolysis resistance of aqueous solutions. The use of 2-propanol (IPA) is clearly distinguished in this approach. Adding 1% IPA has significantly extended the possibility of observing delicate polymers in LC-TEM.^109^ The practical ability to eliminate OH^–^ groups by IPA was also confirmed during the work on synthesizing PdH_*x*_ phases^108^ and polymer nanomotors imaging.^68^ It is worth noting that the issue of scavenging of radiolysis products has also been solved by simulations.^138^ Among other substances that extend the valuable observation time in LC-TEM by scavenging harmful radicals, it is worth mentioning hydroxyapatite,^110^ titanium dioxide^139^ and heavy water (deuterium oxide, D_2_O^140^). It is also worth observing the development of cryoEM techniques, where, for example, the scavenging potential of sodium ascorbate (SA) has been recently noticed.^141^ Some examples of radical scavenger action are summarized in Figure 4. Undoubtedly, using chemically neutral scavengers inside the liquid cell will be a significant step toward taming harmful radiolysis processes and the prospect of conducting TEM observations of viable biological samples in a liquid. However, it is essential to remember that there is a narrow line separating the beneficial limitation of radiolysis products from the potentially harmful effects of the scavenging substance itself, and the topic of ionizing radiation’s influence on electron imaging remains extremely complex.^47^

## 4 Live-Cell Experiments in Literature

Based on the foundations of life, scientists claim that living organisms share seven traits: organic nature, high degree of organization, preprogramming, interaction (or collaboration), adaptation, reproduction, and evolution.^142^ The smallest unit of life appears to be a cell. It possesses the key attributes common to all living things, including: the ability to respire, grow, reproduce, move, metabolize, excrete, and be responsive to the environment. Cells are broadly classified into two main categories: prokaryotic cells (including bacteria and archaea) and eukaryotic cells, represented by fungi, protists, animals, and plants. Considering fundamental differences between the two cell types, in prokaryotic cells, genomic DNA is organized in nucleoids not surrounded by membranes. In contrast, eukaryotes contain chromosome-containing nuclei surrounded by the nuclear envelope. Most eukaryotes also have further membrane-bound organelles, mitochondria, or chloroplasts.

**Figure 5:**
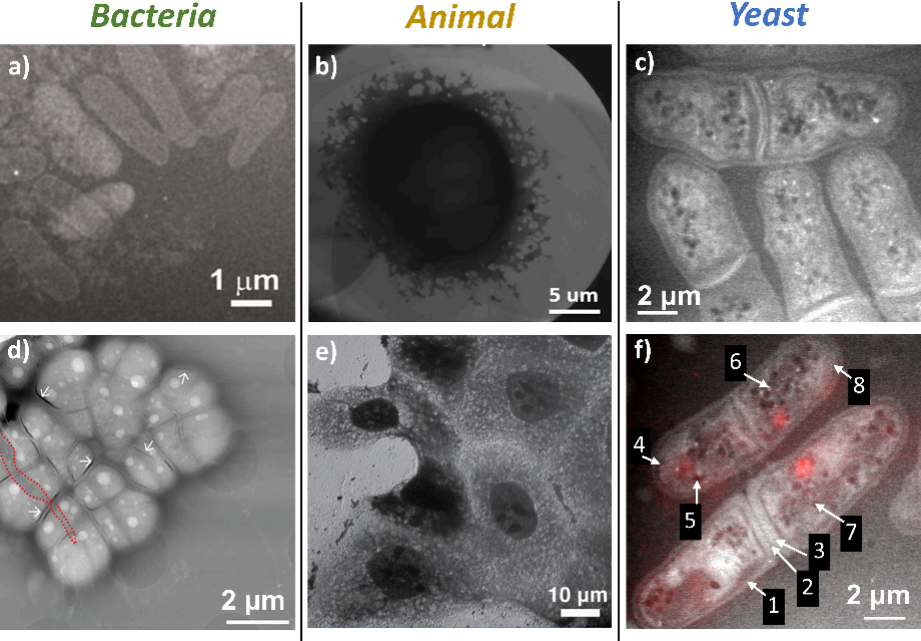
Three main types of cells (bacterial, animal, and yeast) were observed using LC-(S)TEM. a) LC-STEM image of *E. coli* bacteria microcolony stained with 0.1% (w/v) UA/buffer solution. Adapted with permission from ref (23). Copyright 2016 American Chemical Society. (b) LC-TEM image of HeLa cell in a growth medium. Adapted with permission from ref (35). Copyright 2023 Springer Nature. (c) LC-STEM image of Spn3Δ mutants of yeast *S. pombe*. Adapted with permission from ref (37). Copyright 2011 Biophysical Society, Elsevier. (d) LC annular dark-field (ADF) STEM image of *D. radiodurans*. The image shows desiccated cells in a leaky GLC. The dashed red line outlines a tear in graphene, and white arrows indicate areas of membrane separation caused by drying. Adapted with permission from ref (40). Copyright 2024 The Author(s) under CC BY 4.0, published by Wiley-VCH GmbH. (e) LC-TEM image of fixed but hydrated Madin–Darby canine kidney (MDCK) epithelial cells Adapted with permission from ref (11). Copyright 2015 American Chemical Society. (f) LC-STEM image of wild-type *S. pombe* with indicated organelle locations: (1) the cell wall, (2) the primary septum, (3) the secondary septum, (4) a cell membrane invagination, (5) a lipid droplet, (6) a peroxisome, (7) an unclassified vesicle, and (8) a gold nanoparticle (used for focusing). The red color comes from the overlaid fluorescence image, indicating that the cells were alive at that time. Adapted with permission from ref (37). Copyright 2011 Biophysical Society, Elsevier.

The reported live-cell (S)TEM experiments have been conducted on various cell types, including bacterial cells,^7,18−21,23,26,28,29,31,32,36,38−40^ animal cells,^22,30,35^ and a few notable studies on yeast cells^7,24,37^ (Figure 5). Most of the mentioned research was not supported by cell viability studies after electron imaging. However, they will be discussed, as the contribution of these works cannot be omitted. The following section describes live-cell imaging over the years with detailed experimental information on electron dose values, liquid cell type, and cell viability testing. **It is important to emphasize that even though the experiments were labeled as “live-cell”, the cell survivability after electron imaging is still questionable**(24) and will be discussed further in this review.

### 4.1 Bacterial Cells

Due to the relative simplicity, fast multiplication, and motivation of growing antibiotic resistance, bacteria are the subject of most live-cell (S)TEM studies. Before they are described, it is necessary to recall the key differences between the two major groups of these microorganisms. Bacteria are broadly classified as Gram-positive and Gram-negative, according to the differences in cell envelope structure and composition. Gram-positive bacteria have a relatively thick cell wall of cross-linked peptidoglycan (murein), usually the cell’s most external layer. Peptidoglycan is a polymer composed of β-1,4-linked glycans cross-linked by short d-amino-acid-containing peptide chains. Gram-negative bacteria, in turn, have a thin layer of peptidoglycan surrounded by an outer membrane containing lipopolysaccharide (LPS) as a major component. LPS consists of (i) hydrophobic lipid A, (ii) hydrophilic core polysaccharides, and (iii) a hydrophilic O-antigen composed of repeating distal oligosaccharides. LPS devoids the O-antigen is called lipooligosaccharide (LOS).^143^ Gram-negative *Escherichia coli* and Gram-positive *Deinococcus radiodurans* are shown in Figure 5a,d, respectively. A more detailed description of the influence of cell wall structure on cell imaging tests is provided in Sections 4.4 and 5.2.

#### 4.1.1 First Live-Cell Attempts, Method Development, and Bacteria Response to Liquid Cell (S)TEM Environment

The very first attempt for live-cell (S)TEM imaging was performed on Gram-negative bacteria of the species *E. coli*, *Klebsiella pneumoniae* (*K. pneumoniae*), and on simple eukaryotic yeast cells *Saccharomyces cerevisiae* (*S. cerevisiae*) in 2008.^7^ This study was not only the first live-cell experiment but also the first report on (now commercially available^72^) K-kit liquid cell based on prefused two-electron-transparent SiO_2_ membranes. The authors used fluorescence and LC-TEM to perform basic morphological studies of *K. pneumoniae* in a hydrated environment, beam-induced damage, and in situ tellurite reduction.

The next attempt for a live-cell experiment on bacteria was described in 2011.^18^ Working with *Bacillus subtilis*, the authors developed a protein-functionalized GLC dedicated to the observations of Gram-positive bacteria. This specific graphene enclosure helped to decrease cell shrinkage, reduce electron charge, and achieve a higher spatial resolution. The study proved that this type of enclosure prevented morphological changes in microbial cells and increased the quality of their observations. However, viability was not determined after TEM analyses, so the exact impact of graphene on cell survival was not determined.

Another microorganism—*D. radiodurans*—was under study in work from 2012.^19^ This extremophilic Gram-positive bacteria are known as the most radio-resistant organisms in the world that can tolerate extreme environments such as ionizing radiation, oxidation, and desiccation.^144^ The authors demonstrated culture growth on SiN membranes with minimal oxygen access and proposed a way to estimate the microbe thickness using image intensity analysis. The same bacterial species were used in a recent work for a detailed ultrastructure description of cells in GLC.^40^ The cells were imaged with an electron dose of 100 e^–^/nm^2^, which, as the authors stated, was a safe dose for these radiation-resistant species in radical-scavenging GLC. Different cell growth stages were imaged in LP-STEM with detailed energy dispersive X-ray spectroscopy (EDS) elemental analysis. The cell morphology changes due to leaky GLC are shown (Figure 5d). Nevertheless, in both cases, cell viability studies were not performed after LC-STEM imaging, so these highly resistant species’ exact electron radiation tolerance was not demonstrated.

After 2014, a successive increase in research on live-cell subjects can be noticed. In 2015, SiN-enclosed *E. coli* was examined for beam-induced damage.^21^ This time, cell survival was observed using fluorescence imaging and commercial markers, but the exact electron dose was not specified.

A notable work on *E. coli* and P1 bacteriophage was published in 2016, in which the authors used low-dose STEM and, for the first time, established a median lethal electron dose of LD_50_ = 30 e^–^/nm^2^ for these bacteria.^23^ The work was focused on imaging bacteria–bacteriophage interaction with high resolution down to 5 nm with an emphasis on cell survival conditions. An interesting but confusing approach in this work was the use of a low-concentration uranyl acetate (UA) solution to increase the contrast observed in the liquid phase (Figure 5a). UA is a standard dye used for negative staining in TEM^2^ and is generally cytotoxic. Nevertheless, the authors claimed that the stained cells remained alive for low UA concentrations (0.01–0.1% (w/v)) and survived the (S)TEM imaging at an electron dose of 30 e^–^/nm^2^. In this case, the cell viability was checked by fluorescence of standard markers, propidium iodide (PI) and SYTO 9, and cell proliferation assays. However, the results of this research were a subject of controversy in the scientific community.^24^ The critics suggested that the positive result of the LIVE/DEAD assay is not enough to define the cell as a living organism and the reproduction capability under such conditions should be crucial. In this short comment, the authors made some observations on yeast cell *S. pombe*. These results, as well as the definition of a living cell, will be discussed in detail in Sections 4.3 and 5.1.

In the following year, the authors of the mentioned work^23^ responded to the critical comment^24^ from 2016 claiming that the observations of *E. coli* using an electron dose of <29 e^–^/nm^2^ were reliable and the cells survived the electron damage.^27^ Survival was proven by observing the expression of the green fluorescence protein (GFP) gene in irradiated cells, which is claimed to be characteristic only of living microbes.

Further fundamental works on bacteria were performed on Gram-negative bacteria *Cupriavidus metallidurans* (*C. metallidurans*).^38,39^ These works focused on LP-(S)TEM development. A novel multiwindow device was created for better sample imaging, where grid bars function as focusing aids that help get a proper focus without risking creating artifacts. The authors also compared LP-(S)TEM with the cryo-EM technique regarding cell visibility and signal-to-noise ratio differences for thick samples but without viability tests.

In recent years, electron tomography was also utilized for LP-TEM in bacterial cells.^29^ The research showed the possibility of obtaining electron tomography on hydrated cells of the model *Agrobacterium* host and flagellotropic phage with a cumulative electron dose of 1000 e^–^/nm^2^. Despite exceeding typical lethal doses, the further development of this method could be advantageous in detailed biological interactions research. However, the time resolution and low electron dose regime might be challenging to overcome.

#### 4.1.2 Biomineralization Processes in Living Magnetotactic Bacteria

Biomineralization is a natural process in which living organisms obtain chemical elements from the surrounding environment and convert them into minerals. This process occurs, for example, in magnetotactic bacteria *Magnetospirillum magneticum* (*M. magneticum*), which produces magnetosomes.^145^ Magnetosomes are intracellular structures formed by magnetite (Fe_3_O_4_) or greigite (Fe_3_S_4_) nanocrystals surrounded by a phospholipid membrane. These magnetic nanocrystals are typically organized in chains and allow bacteria to reorient in a magnetic field.^146^ The exact magnetosome biomineralization mechanism is still unknown;^147^ therefore, the live-cell approach has gained scientific interest in studying this process.

**Figure 6:**
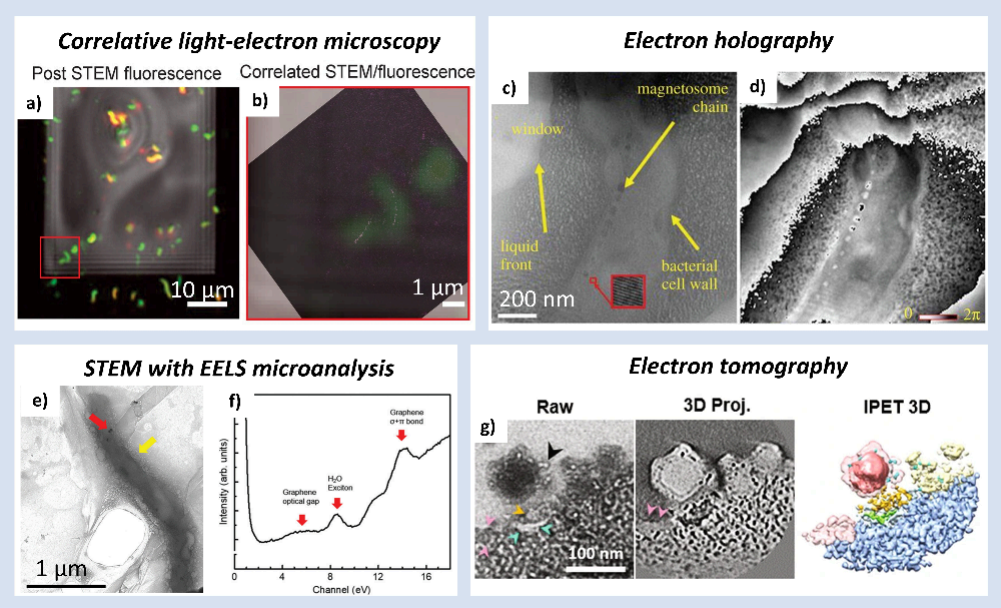
Advanced (S)TEM methods used in live-cell research. (a, b) Correlative light-electron microscopy on magnetotactic *M. magneticum* cells stained with PI and Syto9 dyes. The merged image (b) comes from the area indicated by the red rectangle in the left (a) picture. Adapted with permission from ref (20). Copyright 2014 The Author(s) under CC BY-NC-ND 4.0. (c, d) Electron holography on *M. magneticum* bacteria. TEM image (c) shows characteristic features of the sample: bacterial cell wall, magnetosome chain (magnetic nanocrystals), liquid front, and the window (blank area). The corresponding reconstructed phase image (d) of the same cell. Magnetosomes are visible in the phase image. Adapted with permission from ref (26). Copyright 2017 The Royal Society. (e, f) (S)TEM with EELS analysis performed to analyze the graphene enclosure and magnetosomes imaging on *M. magneticum* bacteria. The bright-field TEM image (e) shows a bacterium in GLC. The red arrow points to magnetosomes (high-contrast nanocrystals), and the yellow arrow indicates the bubbling induced by the electron beam. Fingerprints for graphene and water in low-loss EELS microanalysis (f) show effective bacterial liquid enclosure in GLC. Adapted with permission from ref (28). Copyright 2019 The Royal Society of Chemistry. (g) Electron tomography of lentiviral vectors interacting with HeLa cell membrane. The images present: tilted TEM image (Raw), contour enhanced projections (3D Proj.), and individual particle electron tomography (IPET 3D) reconstruction. Adapted with permission from ref (35). Copyright 2023 The Author(s) under CC BY 4.0.

The first documented in situ observations on *M. magneticum* date back to 2014, when the authors used a correlative light-electron microscopy approach.^20^ The strong contrast from magnetosomes inside the cells allowed reliable STEM observations and beam damage analysis, with restricted electron dose exposures ranging from 24 to 72 e^–^/nm^2^. The damage in the structure of bacteria was visible for the cumulative electron dose above 100 e^–^/nm^2^. Despite the positive fluorescent test, the cells showed no life processes, such as reproduction or enzymatic activity. This novel work showed an approach to obtain insight into cell viability after STEM imaging with highly controlled electron dose focusing on fluorescent signal reliability. The effects of the correlations are presented in Figure 6a.

Another interesting approach for live-cell (S)TEM imaging on *M. magneticum* is off-axis electron holography (Figure 6b).^26^ This field-sensitive technique easily distinguished the magnetic field of magnetite nanocrystals inside the bacteria. The authors kept the low electron dose of 10 e^–^/nm^2^, but the surrounding liquid changes (bubbling) were still visible. Despite the challenges of using a reference beam near the area of interest, this technique holds significant potential for advancing LP-(S)TEM studies, including those focused on biological interactions. However, in this case, bacteria were not checked for any living properties; therefore, their survival after holographic imaging remains unknown.

The same species of *M. magneticum* were the subject of another magnetite biomineralization study two years later with GLC enclosure of the cells.^28^ Using graphene instead of SiN (utilized in almost all research mentioned before) allowed for more resolved imaging of magnetosomes down to 10 nm and their detailed chemical composition analysis. Additionally, the effectiveness of the graphene enclosure was checked by using very sensitive electron energy loss spectrometry (EELS), which indicated the presence of both water and graphene (Figure 6c). The authors stated that the cells were viable after TEM imaging, which was proved by fluorescence imaging and the fact that the biomineralization process could occur only in a living organism and its progress was observed in situ.

#### 4.1.3 Antimicrobial Actions

The increasing use of antibiotic therapy has led to new strains of bacteria resistant to this treatment. Hence, the different methods for curing microbial infections, based on the antimicrobial actions of photosensitizers^148^ and nanomaterials^149^ have gained the attention of scientists. Subsequently, some reports on the subject can be found.

Starting in 2021, the live-cell approach was used in antimicrobial photodynamic therapy studies on bacteria enclosed using carbon films.^31,32,36^ The authors used a custom setup for light illumination of *Staphylococcus aureus* (*S. aureus*) in photosensitizer to observe the mechanism of bacteria inactivation in situ. In this case, the electron dose was relatively very high (on the order of 5000 e^–^/nm^2^), and the cells’ death was assumed. Another study on antimicrobial properties was conducted on *Acetobacter aceti* with gold nanoclusters.^33^ The cells were placed in a commercial SiN K-kit.^34^ The liquid enclosure and reliability of this study found some concerns as the cells did not look surrounded by liquid.^34,150^ These studies did not check cell viability, and the electron dose was not specified. Therefore, even though the experiments started on living cells, the exact viability was not specified, and the cells were presumably dead after imaging, as the electron doses were very high in these studies. Anyway, the live-cell approach seems promising for future studies in the subject.

### 4.2 Animal Cells

#### 4.2.1 Fixed Animal Cells in a Hydrated State

As mentioned earlier, LP-TEM imaging causes many problems and the cells may undergo a substantial morphological change upon electron irradiation. When the experiment does not require in situ observations on living cells, they can be fixed. Thanks to this, their structure is well preserved with higher contrast and the specimen is less sensitive to electron irradiation. This makes higher-resolution imaging easier than that in living cells, allowing the observation of single membrane proteins. Although fixed, to obtain results close to the living state, the cells must remain hydrated so the specific components (like cell membranes) keep the native structure. Therefore, observations on fixed cells performed by LP-(S)TEM cannot be counted as live-cell research. Nevertheless, we highlight these works’ importance in electron microscopy research on cells, comparison with super-resolution microscopy methods, and as an excellent example of correlative light-electron microscopy applications.

The first reports on animal cell studies appeared in 2009.^9^ The cells used in the research were not living but fixed and kept hydrated. COS7 cells (African Green Monkey fibroblast) were labeled with 10 nm gold nanoparticles (for STEM imaging) and quantum dots (for confocal fluorescence imaging). The authors compared the obtained STEM and confocal microscopy results with the resolution of known super-resolution techniques: stimulated emission depletion (STED), photoactivated localization microscopy (PALM), and stochastical optical reconstruction microscopy (STORM). The results showed a higher possibility of imaging individual proteins using electron microscopy (with 80 nm resolution) than the light-based ones. The physics of LP-STEM imaging is also described in detail in this work. In later years, the same group combined environmental scanning electron microscopy (ESEM) imaging with the STEM detector for observations of SKBR3 cells (human breast cancer cells) enclosed on SiN with graphene.^10,12^ Another work on hydrated SKBR3 cells dates to 2017.^17^ The authors managed to obtain a very high resolution of 2 nm, thanks to hybrid liquid cell enclosure consisting of a SiN membrane on the bottom and graphene sheet on the top. This allowed them to distinguish ErbB2 protein monomers from dimers in whole cells. For the correlation with fluorescence microscopy, quantum dots were used as the labeling agents for the studied proteins. The observations were performed in a wide range of electron doses starting from 1000 e^–^/nm^2^. This work describes how graphene can help achieve high resolutions so that a single protein can be distinguished within relatively thick cells. In another work, fixed Madin–Darby canine kidney (MDCK) epithelial cells (Figure 5e) were used for the development of graphene enclosure^11^ as described earlier in Section 2.4 of this review.

#### 4.2.2 Living Animal Cells

As mentioned in the previous paragraphs, a notable increase in experiments on cells in 2015 can be observed, and this year, a first “live-cell” report on animal cells can be found.^22^ This study’s subjects were NOTCH1-positive glioblastoma stem cells interacting with gold nanorods, where SiN microchips were etched to obtain microwells for further liquid enclosure. The electron dose in this case was relatively low, reaching 50 e^–^/nm^2^, but cell viability remained unknown. The researchers claimed to observe the dynamics within the whole cell, which gave the foundation for further understanding and application in nanomedicine.

In 2019, another live-cell research was performed on MIN6 β-cells.^30^ The LC-STEM study focused on insulin granule dynamics (fusion and exocytosis) and was supported by fluorescence microscopy, EDS, and EELS. This work presents a detailed cell viability analysis starting from the 6 h cell incubation in a GLC without electron irradiation and continuing after TEM studies. After 2 h of imaging under low electron dose conditions, 73% of the cells were still viable. The loss of viability due to electron irradiation was calculated to be relatively low with a value of only 18%. The electron dose was kept under 100 e^–^/nm^2^.

In the most recent report on animal cells from 2023, a new, very accessible liquid cell enclosure technique based on two Formvar-coated grids was presented.^35^ In this research, HeLa cells (Figure 5b) were mixed with a virus-like lentiviral protein transfer vector, and their interactions were studied using LP-TEM and electron tomography (Figure 6d). This approach helped us visualize the subsequent stages of virus cell entry. Although this research was not supported by any cell viability studies, it demonstrated an alternative way for presumed “live-cell” experiments with total low electron dose of 100 e^–^/nm^2^.

### 4.3 Yeast Cells

Only three reports on yeast live-cell research can be found in the literature.^7,24,37^

The first one was already mentioned in the subsection about bacterial cells as the very first attempt at live-cell imaging in 2008.^7^ The researchers used two bacterial species and *Saccharomyces cerevisiae* (*S. cerevisiae*) yeast cells. Nevertheless, this work only shows the basic, nonquantitative fluorescence results for yeast species.

Another report on living yeast cells came a few years later in 2011.^37^ A common rod-shaped, unicellular, eukaryotic model organism *Schizosaccharomyces pombe* (*S. pombe*) of wild-type and its Spn3Δ mutant (Figure 5c) were used to demonstrate LC-STEM for yeast cells structure distinction. A very detailed organelle location was obtained without any fixation or labeling (Figure 5f). The results showed the advantage over light microscopy in imaging a living cell’s ultrastructure by achieving an order of magnitude higher magnification. The average electron dose used in this study was 22 e^–^/nm^2^, and the authors stated that the cells were viable only at the beginning of electron imaging and did not survive afterward. A few years later, the same authors used *S. pombe* as a model to prove that STEM imaging, even at low electron dose conditions of 20 e^–^/nm^2^, ends with cell death.^24^ This work was already mentioned in Subsection 4.1.1, as it critically addressed other live-cell research on bacteria.^23^ Cell viability was tested with a standard (for yeast) FUN-1 fluorescent dye, and morphological changes were observed in STEM. This concise work concluded that live-cell (S)TEM imaging is “probably impossible”.

### 4.4 Summary

The literature contains more than 20 live-cell reports. These works are a profound foundation for future live-cell development, which can include in situ observations, but its limitations need to be faced first. The examples cited indicate that **most studies using the live-cell approach have either not assessed the viability of the bacterial objects under study or have done so in a manner that raises numerous doubts**.

First, fluorescence microscopy specified a general lethal electron dose for cells only for *E. coli* bacteria^23^ at LD_50_ = 30 e^–^/nm^2^. However, further work on yeast *S. pombe* cells^24^ undermined this relatively small value, where 20 e^–^/nm^2^ was enough to terminate the cells. This raised the question of whether any live-cell studies were performed on living organisms. Further study^27^ determined an even lower dose of LD_50_ = 10 e^–^/nm^2^ based on the cell’s ability to express GFP, of which fluorescence signal was analyzed after LP-STEM. Anyway, these values were determined only for two cell species of different types (bacterial and yeast), which, in our opinion, is very general, and the exact LD_50_ values can be specific for distinct microorganism species and dependent on imaging type (different in TEM, STEM, and specific liquid cell types).

A variety of cells were studied: bacterial (*E. coli*, *K. pneumoniae*, *B. subtilis*, *D. radiodurans*, *C. metallidurans*, *M. magneticum*, *Agrobacterium* sp., *S. aureus*, and *Acetobacter aceti*), animal (glioblastoma stem cells, MIN6 β-cells, and HeLa cells), and yeast (*S. cerevisiae* and *S. pombe*). No systematic study was conducted on these species’ differences (especially in electron imaging survival). For example, the lethal electron dose for highly resistant Gram-positive *D. radiodurans* could be studied in detail and compared to those of other known bacteria. Instead, the “safe” electron dose for these species in GLC was estimated to be 100 e^–^/nm^2^ based only on the fact that they are more resistant to radiation than Gram-negative *E. coli* without any comparative study.^40^ The structural differences between Gram-positive and Gram-negative bacteria affect the distinct bacteria’s vulnerability to environmental factors or antimicrobials. Accordingly, Gram-positive bacteria, surrounded by thick cell wall, are generally more tolerant to desiccation, UV and γ irradiation, temperature stress, and ionized gas-containing cold atmospheric plasma but less tolerant to pH variations.^151−153^ In contrast, due to their distinctive structure and additional surface asymmetric lipid bilayer (outer membrane), Gram-negative bacteria are more resistant than Gram-positive ones to current antibacterial agents, including antibiotics and disinfectants^154^ or antibacterial methods utilizing photodynamic inactivation.^155^ Since specific differences in bacterial cell envelope structure are those associated with varying resilience to adverse environmental stressors, they are also crucial for microscopic examinations in terms of selecting the most suitable conditions.

The highest electron dose which (as stated by the authors) did not cause cell death^28^ was 200 e^–^/nm^2^. In general, in the studies that claimed cell survival after LP-(S)TEM imaging, the dose did not exceed 100 e^–^/nm^2^. The experimental details of these studies are presented in Table 2 in Section 5.3. The primary technique used for survival studies was fluorescence microscopy, which does not always indicate the exact cell viability and instead gives information about the cell membrane or DNA state (damaged or intact), so the positive signal may come from the dead cell anyway. A few studies^27,28^ stated that some processes (biomineralization, gene expression) occur only in living cells; thus, the observations were performed on viable cells. This gives an alternative for determining cell viability, but in our opinion, the more living activities observed in the cell, the more accurate the estimation is, as we discuss in Section 5.

Another issue connected to live-cell studies is the fact that many (especially early) studies determined the cell damage based on its morphology.^7,18,21,38^ Visible damage usually appears long after the cell is terminated by an electron beam; therefore, this approach is inaccurate. For the same reason, observations of antimicrobial actions on living cells need to be performed with awareness and reliable control experiments.

To sum up, most live-cell studies lack reliable viability testing after LP-(S)TEM imaging, and in some of them (including recent ones), the electron dose is not specified. Therefore, we believe most of them may not show results on living organisms, and the cells’ fate remains somewhat unknown. Nevertheless, these studies are crucial, as they lay a profound foundation for developing many aspects of live-cell imaging. It should be emphasized that the live-cell technique is very demanding regarding sample preparation, imaging, analysis, and interpretation, so more on this subject is yet to come.

## 5 Methods for Cell Viability Verification: Fluorescence Microscopy

### 5.1 General Criteria of Cell Viability

In general, there are three recognized criteria for verification of cell viability, including the following: (i) culturability referring to the ability to grow in a relevant environment, (ii) metabolic activity referring to the variety of cellular biochemical processes, and (iii) cell membrane integrity relating to disrupted and/or broken membrane in death cell while an intact membrane in living cells.^156^ While assessing viability in mammalian cells seems more unequivocal for microorganisms, obtaining reliable confirmation of viability is not always possible based on a single criterion. As summarized in Table 1, *while one criterion is usually sufficient to confirm a cell is alive, using two criteria to determine its death reliably is generally safer.* For instance, live bacteria in unfavorable environmental conditions can enter a nonculturable state, such as in the case of VBNC (viable but nonculturable) bacteria, where they maintain low metabolic activity and lose the ability to divide.^157^ Furthermore, viable but nonculturable bacterial pathogens may exist or even remain in a metabolically inactive state, a so-called dormant or persistent state, for years.^158^ Key examples of nondividing persisters are bacteria that exhibit multidrug tolerance, enabling them to survive the treatment with all known antibiotics.^159^ This represents a common adaptive strategy observed in many bacterial species, allowing them to withstand environmental stressors. Another example can be bacterial endospores (nonreproductive forms of bacteria produced within vegetative cells by genus, e.g., *Bacillus* or *Clostridium*), which are metabolically inactive yet represent the most resistant forms of life on earth. Endospores, which regain viability by germination under favorable conditions, enable certain bacteria to survive environmental assaults for thousands, if not millions, of years.^160^ These findings challenge traditional definitions of microbial viability, emphasizing the need for multicriteria assessment methods to distinguish between genuinely nonviable cells and those in a metabolically active yet nondividing state (as in the case of VBNC). By analogy, microorganisms that lack metabolic activity can still be either dead, like vegetative cells, or alive but dormant, like endospores. Relying solely on membrane integrity can also be misleading in the assessment of microbial viability. For example, UV-killed bacteria with DNA damage following brief radiation exposure are not metabolically active but still may retain intact membranes.^156^ Thus, as demonstrated by these examples, each criterion of viability has limitations; when used separately, it can sometimes lead to false conclusions. To mitigate these limitations, it is necessary to refine the concept of viability and, in cases of uncertainty, to use more than one method for its determination. More information on the death/viability topic the reader can find in a review by Trinh and Lee from 2022.^161^

**Table 1:** Microbial Viability Interpretation Based on the Selected Assessment Criteria

Criterium of viability	Exist	Not exist
culturability	alive	alive or dead
metabolic activity	alive	alive or dead
membrane integrity	alive or dead	dead

Considering the discussed criteria for cell viability, its determination in the laboratory relies on assessing cells’ various metabolic and structural properties. Accordingly, culture-based methods, including colony counts on appropriate media or optical density (OD) measurements of exponentially growing bacteria, are commonly used to evaluate culturability. While the visible growth of bacterial colonies confirms viability, the viability assessment, based on optical density, should always be performed by using bacteria from the logarithmic growth phase. This is because nonlysed dead and viable cells can have similar OD readings, making it an unreliable indicator outside active growth conditions. Metabolic activity, in turn, can be evaluated by measurement of enzyme activity, coenzyme production, or nucleotide uptake activity etc.^162^ Finally, membrane integrity is usually evaluated by assessing membrane permeability using either dye exclusion methods or enumerating fluorescently labeled bacteria by fluorescence microscopy or flow cytometry.

### 5.2 Microorganisms as the Best Model for Assessing Viability after Ionizing Radiation Exposure

The killing of cells by radiation generated in an electron microscope is still one of the significant challenges of live-cell imaging. Even though microorganisms are irreplaceable in research, where survival in extreme conditions is desired, it is not easy to find a universal or straightforward correlation between the vulnerability of cells to an electron beam and their cellular and physicochemical properties. This suggests that the sensitivity of living organisms to electrons is determined by a set of their intrinsic cellular features in which the superimposition of many physicochemical, structural, and molecular properties exists.

In comparison to animal cells, bacteria show higher resilience to electron doses due to several unique features they possess including the following: (i) peptidoglican-containing cell wall that provides a barrier and higher resistance to structural degradation and prevents osmotic lysis,^163^ (ii) the lack of highly sensitive to radiation membrane-surrounded organelles such as mitochondria, endoplasmic reticulum, or nucleus, which can be easily damaged through lipid bilayers peroxidation leading to membrane rupture and organelle dysfunction,^163^ (iii) less hydrated cytoplasm, which decreases radiolysis simply by having fewer water molecules available for it and weaker generation of reactive oxygen species (^•^OH, H_2_O_2_) responsible for breaks of DNA strands, oxidation of proteins and peroxidation of lipids, (iv) much higher concentration of macromolecules in the cytoplasm (∼55% (w/w) proteins, ∼15% (w/w) rRNA) and in membranes known as macromolecular crowding; this phenomenon has clear impact on the mobility of molecules limiting their diffusion but possibly also limiting the diffusion of smaller units of atom—electrons.^164,165^

Referring to Table 1, the most definitive confirmation of microbial viability following radiation exposure is the ability to undergo cell division. To date, culturability has been documented, as described earlier in this manuscript, in radioresistant bacteria *D. radiodurans*, displaying approximately 90% survival on agar plates following exposure to 5.2 kGy of γ radiation.^166^ This finding of viability among bacteria irradiated during the exponential phase of growth, when they are most sensitive to any stressors, highlighted the existence of a tremendous tolerance of certain microorganisms to ionizing radiation. Similarly, for nonradioresistant bacteria such as *E. coli* and *B. subtilis*, which lack robust mechanisms mitigating oxidative stress, culturability was observed, however, only under specific conditions—notably, after higher but not lethal electron exposure during the acquisition of high-resolution SEM images, but exclusively in graphene-covered cells.^137^ This protective role of graphene in preserving cell integrity and function raises intriguing possibilities for its use, where microbial survival in extreme conditions is required. Interestingly, *E. coli* cells exposed to lethal doses of electron beam radiation (7.0 kGy) admittedly lost their replication capability but still retained their ability to propagate bacteriophages, maintained intact membranes, and were metabolically active for up to 9 days postirradiation. This suggests that lethally irradiated *E. coli* cells, despite their loss of division potential, continue to temporarily function at a manner resembling rather live nonirradiated cells than thermally killed (dead) cells.^167^

Although methods based on culturability are the most unambiguous for assessing cell viability, their usage after LC-S(TEM) imaging is still limited because of technical problems such as low bacterial load in the sample and the fact that usually only a few cells are being irradiated by an electron beam during the whole experiment. In the case of SiN liquid cells prepared in a dedicated holder, the microfluidic chamber can be disassembled after imaging, so the cells can be placed back into a rich growth medium and incubated with vigorous shaking. However, to obtain culturability results, the viability experiment would have to be performed on at least 50–100 cells (CFU-colony forming units), which is a large number for observations on one liquid cell sample. Disassembly would be more difficult or even impossible in the case of all other liquid cell types (for example, when graphene wraps whole bacteria irreversibly).

### 5.3 Fluorescence-Based Microscopic Methods As the Gold Standard in Live-Cell (S)TEM Experiments

Among the numerous well established cell viability methods,^162^ some of them are quite simple and require less complicated measuring tools such as a light or fluorescence microscope. In contrast, others require high-quality measurement equipment, including a spectrophotometer, fluorometer, chemiluminometer, or flow cytometer. Based on the type of equipment used, these methods are commonly categorized as microscopic, colorimetric,^168^ fluorometric,^169^ luminometric,^170^ and flow cytometric methods.^171,172^ As evidenced by the examples cited, various approaches are available. Since the purpose of this work is not to describe the broad methodology used in assessing cell viability but only those aspects of it that can be used in the study of the live-cell approach, in the remainder of this review, we will focus on just this issue.

**Figure 7:**
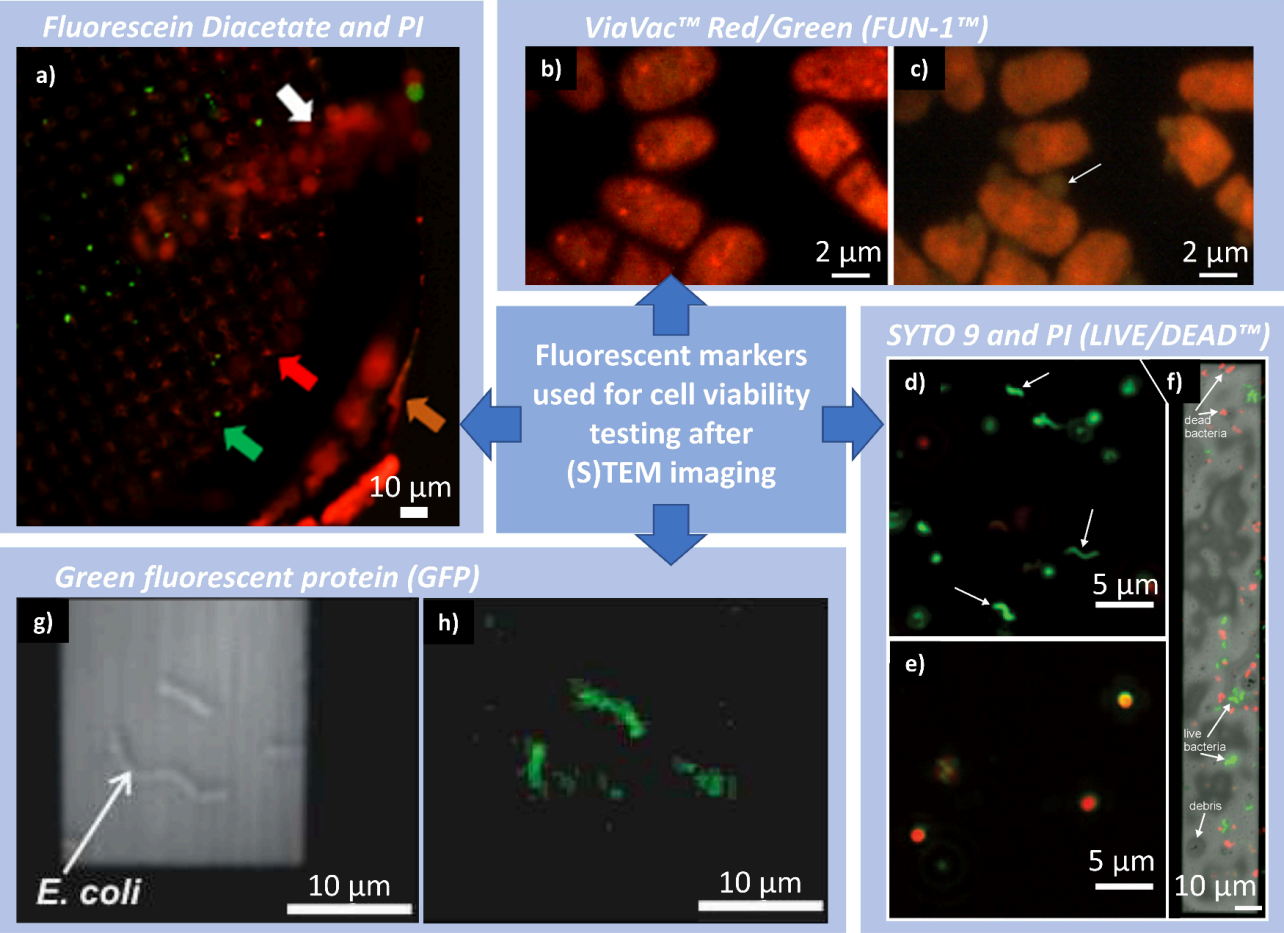
Fluorescent images of the cells before and after (S)TEM imaging using standard fluorescent dyes. (a) Fluorescein diacetate (FDA) and propidium iodide (PI) used to determine the MIN6 β-cells viability after 6 h incubation in GLC. Adapted with permission from ref (30). Copyright 2019 The Author(s) under CC-BY-NC 3.0. (b, c) Yeast cells imaged using FUN-1 dye where the panel b image presents living cells before electron imaging (bright red signal) and panel c shows dead cells (orange-yellow fluorescence) after taking two STEM images at a dose of 20 e^–^/nm^2^. Adapted with permission from ref (24). Copyright 2016 American Chemical Society. (d–f) *M. magneticum* cells stained with PI and Syto9 dyes. In panel d, the arrows indicate the cells with inact cell membrane (green fluorescence); panel e shows the bacteria killed with isopropyl alcohol (red fluorescence); and panel f shows the fluorescent signal from bacteria in SiN liquid cell for STEM imaging. Both living (green) and dead (red) cells were present in the sample. Adaptedwith permission from ref (20). Copyright 2014 The Author(s) under CC BY-NC-ND 3.0. (g, h) *E. coli* viability measured by GFP gene expression after STEM imaging. In panel g, light microscopy image shows the bacteria in a liquid cell, and in panel h is shown the corresponding fluorescence signal of GFP. Adapted with permission from ref (27). Copyright 2016 American Chemical Society.

In all live-cell LC-(S)TEM reports that tested the cell viability, fluorescence microscopy was a method of choice, as it is relatively easy to implement in a specific area of (S)TEM imaging. The resolution of this technique can be increased by using confocal microscopy;^173^ thus, both microscopic techniques can be easily correlated. In the S(TEM) studies mentioned in the previous paragraph, mainly well established assays and commercial cell viability kits were used. All of the recent reports are summarized with the experimental details in Table 2, and the examples of fluorescence imaging on (S)TEM samples are presented in Figure 7.

**Table 2:** Chronological List of Live-Cell Experiments in Which the Authors Checked the Cell Survival after (S)TEM Imaging^a^

Cell type	Cell species	Estd survival after (S)TEM	Fluorescent markers	Electron dose (e^–^/nm^2^) or electron dose rate [e^–^/(nm^2^s)]	Liquid cell type	Year	Ref
bacterial, yeast	*Escherichia coli*, *Klebsiella pneumonia*, *Saccharomyces cerevisiae*	alive	PI and Syto-9	10^–6^ e^–^/(nm^2^s)	SiO_2_ membrane based (first K-kit)	2008	(7)
yeast	*Schizosaccharomyces pombe*	dead	FUN-1	22 e^–^/nm^2^	SiN membrane based (liquid flow holder, Protochips, Raleigh, NC)	2011	(37)
bacterial	*Magnetospirillum magneticum*	not specified: membrane intact but other properties (reproduction, enzymatic function) not observed	PI and Syto-9	10 e^–^/nm^2^	SiN membrane based (liquid cell holder, Hummingbird Scientific, Lacey, WA, USA)	2014	(20)
bacterial	*Escherichia coli*	alive	PI and Syto-9	3 × 10^–6^ e^–^/(nm^2^s)	SiN membrane based	2015	(21)
bacterial	*Escherichia coli*	alive	PI and Syto 9	>30 e^–^/nm^2^	SiN membrane based (Poseidon 210 in situ liquid cell TEM flow holder, Protochips Inc., Morrisville, NC, USA)	2016	(23)
yeast	*Schizosaccharomyces pombe*	dead	FUN-1	20 e^–^/nm^2^	SiN membrane based	2016	(24)
bacterial	*Escherichia coli*	alive	GFP	<29 e^–^/nm^2^	SiN membrane based (Poseidon 210 in situ liquid cell TEM flow holder, Protochips Inc., Morrisville, NC, USA)	2017	(27)
bacterial	*Magnetospirillum magneticum*	alive	PI and Syto-9	200 and 2000 e^–^/nm^2^	GLC	2019	(28)
animal	*Endocrine MIN6 pancreatic β-cells*	alive (73% of cells)	cell counter: trypan blue, fluorescence: FDA, PI	<100 e^–^/nm^2^	GLC	2019	(30)

a Abbreviations: PI, propidium iodide; GFP, green fluorescence protein; FDA, fluorescein diacetate; GLC, graphene liquid cell.

#### 5.3.1 Fluorescently Tagged Bacteria

Many strategies use fluorescent dyes to discriminate between live and dead bacteria. An overview of recent advances in these strategies is excellently described in the review by Yoon et al., 2021.^174^ These strategies include specific interactions with bacterial cell wall components, peptidoglycan synthesis reactions, and intracellular enzyme reactions. Although many fluorochromes label bacteria, only a few are used in routine work. For example, for monitoring the viability of bacteria based on membrane integrity, two popular fluorochromes are commonly used: green fluorescent dye (SYTO9) to visualize living cells with intact membranes and red fluorescent propidium iodide (PI) to visualize death or dying cells upon disruption of cell membrane integrity.^175^

Another technique for studying the viability of diverse bacterial species is to incorporate fluorescent probes such as fluorescent d-amino acids (FDAAs) into live bacteria’s cell walls at active peptidoglycan biosynthesis sites. Depending on the chemical structure, the light emitted by FDAA can be blue, green, or red. Significantly, FDAAs facilitate specific probing of bacterial growth without causing significant perturbation^176^ and are suitable for use with confocal and super-resolution microscopy. Yet another illustrative strategy is measuring the gene expression in bacteria using bioluminescent jellyfish *Aequorea victoria* green fluorescent protein (GFP)-based reporter system.^177^ In microbiology, this strategy may be beneficial for marking microorganisms that are difficult to stain with external dyes or for studying the growth kinetics of microorganisms and monitoring the real-time fluorescence. This technique uses the GFP of a genetically engineered strain as a quantitative reporter to visualize gene expression and protein subcellular localization. The significant advantages of GFP, in addition to its ease of detection after irradiation with blue or near UV light and without the need for any exogenous substrates, are its high stability and ability to analyze cells non-invasively.^177^ To date, GFP-based constructs have been successfully used in viability testing of both planktonic and grown-in biofilm Gram-negative bacteria such as *E. coli*(178) and *Pseudomonas aeruginosa*(179) as well as Gram-positive bacteria such as *Enterococcus faecalis*.^180^ Likewise, FUN-1 is a fluorescent dye used in studies of yeast and other fungi to monitor cell viability.^181^

#### 5.3.2 Fluorescently Tagged Animal Cells

In the case of visualization of viability among animal cells, the luminescent-based measurement of ATP using firefly luciferase is a widely used method for estimating viable cell numbers based on membrane integrity. When cells lose membrane integrity, they lose the ability to synthesize ATP, and endogenous ATPases rapidly deplete any remaining ATP in the cytoplasm. In this assay, ATP and luciferin act as substrates for luciferase, resulting in light production. The ATP assay is not only the sensitive method for assessing cell viability but also one of the fastest, as the luminescent signal stabilizes within 10 min after reagent addition, providing a rapid and reliable readout.^182^ A drawback of this method is the requirement for a luminometer to detect the luminescence. Another standard nonfluorescent vital marker is trypan blue, which enters the cell through the porous membrane of dead or damaged cells, staining them blue, but does not penetrate undisturbed cells.^169,183,184^ Likewise, MTT [3-(4,5-dimethylthiazol-2-yl)-2,5-diphenyltetrazolium bromide] colorimetric assay has gained tremendous popularity in assessing the metabolically active, therefore alive, mammalian and yeast cells.^185−187^ Although both methods are commonly used since they are cheap, fast, and easy, the most suitable in S(TEM) imaging are still fluorescently based methods. Analogously to bacteria, animal cells are often stained using a multiparametric fluorescence system, which in the latter makes it easier not only to distinguish between dead or living cells but also that seems to be even more important, to distinguish between different types of cell death, namely, necrosis and apoptosis (programmed cell death). This system is essentially based on two fluorochromes, one of which is green (Alexa Fluor 488, fluorescein isothiocyanate FITC, or SYTOX Green) and the other red (PI or allopycocyanin APC), which can work in different combinations. The most commonly used combinations are Annexin V-Alexa Fluor 488 and PI; Annexin V-FITC and PI; Annexin V-APC, and SYTOX Green. In these systems, one fluorochrome is conjugated to Annexin V, responsible for Ca^2+^-dependent binding of phosphatidylserine residues exposed on the cytoplasmic membrane of apoptotic cells. In contrast, the second fluorochrome is typically used for nucleic acid stains that label dead/necrotic cells lacking cellular membrane integrity.^188^ Yet another commonly used fluorescent tag that discriminates live from dead cells based on plasma membrane integrity is cell-permeable calcein-AM (acetoxymethyl ester of calcein), a nonfluorescent compound that passively enters the cell. Once inside, it is converted by cytosolic esterases into green fluorescent calcein, which is retained by live cells with intact membranes.^189^

In addition to organic fluorescent dyes, inorganic fluorescent nanoprobes are also used in cell studies. Recently, nanoparticle (NP)-based inorganic fluorescent probes, such as dye-containing silica NPs (SiNPs), quantum dots (QD), and metal nanoclusters, have been useful for cell staining and visualization. Their potential for biodetection and cellular/subcellular bioimaging encompasses applications such as membrane imaging, mitochondria visualization, enzyme tracking, and nucleus imaging, making them suitable for live-cell imaging. Inorganic fluorescent probes exhibit many improved optical qualities desirable for biological applications such as high photostability, long fluorescence lifetime, robust signal strength, and broad absorption and narrow emission spectra. Furthermore, they are advantageous for both single-color and multicolor experiments.^190,191^

#### 5.3.3 Specificity of Fluorochromes

Another essential property of fluorophores is their selectivity/specificity. From the biological point of view, dye selectivity refers to the specific chemical interaction between the fluorescent probe and the recognized target (e.g., probe specific to LPS, probe specific to DNA). According to generally accepted laboratory rules, high specificity is a probability that a positive reaction will *not* occur if a specimen is a true negative and vice versa. In other words, a high specificity protects against false-positive responses. This is particularly important in fluorescence-based research, which has the disadvantage of the ability to generate a high background. Therefore, selectivity (although it can) does not necessarily bear the hallmarks of a distinction between what is alive and what is dead. For example, LPS from alive or dead bacteria and even LPS-containing bacterial outer membrane vesicles will be tagged by an LPS-specific probe basically in the same manner without any distinction. Therefore, depending on the research needs, dye selectivity sometimes must be put in a much broader context within the existing and above-described viability criteria. Accordingly, it can be considered: (i) in the context of general identification of living and dead bacteria, which can be used, for example, to assess the effectiveness of antimicrobial compounds or influence of other chemical/physical factors, and (ii) in the context of selective-differential identification and analysis of bacterial populations to discriminate them in a given sample.

#### 5.3.4 Photophysical Properties of Fluorochromes Used in Live-Cell S(TEM)

As mentioned earlier, fluorescence microscopy seems to be the most suitable technique for assessing microbial cell viability after (S)TEM imaging, as it allows checking the exact imaging area. The markers used previously in live-cell (S)TEM research are standard, commercially available fluorochromes (Table 2, Table 3): fluorescein diacetate (FDA) (Figure 7a), FUN-1 (Figure 7b), propidium iodide (PI) and Syto-9 (Figure 7c), and green fluorescent protein (GFP) (Figure 7d), that were used with established protocols.^162,181,192,193^ Thanks to their physicochemical and optical properties, they are easy to use in standard laboratory setups and the results are straightforward to analyze. Most of these fluorochromes have an excitation maximum in the visible light range near 480–490 nm, corresponding to blue and green light, which decreases the possibility of damaging the cells with irradiation of higher energy, such as ultraviolet (UV) radiation. GFP is the fluorochrome, whose excitation maximum is in the UV range (385 nm), but its position (as well as emission maximum) strictly depends on the type (mutant) of used protein and can shift significantly.^194^ The quantum yields of most fluorescent dyes are already high (nearly 0.80 for PI and GFP, and higher than 0.90 for FDA) in the solutions or are significantly increased after binding with specific molecules (like nucleic acids in the case of Syto-9 dye) or depend on metabolic states of the cell (FUN-1). The fluorochromes utilized in live-cell (S)TEM research were used either in pairs (PI and Syto-9 or PI and FDA) or alone (GFP, FUN-1). This helps distinguish between living and dead cells, as the dead cell indicator (PI) emits red light that can be easily distinguished from green light by a human without any additional detectors. Similarly, in the case of GFP and FUN-1, the signal was present or not, which was also convenient to implement in small (S)TEM investigation areas. Of course, the use of specific fluorochromes depends on the experimental specification and the cell type, as described in the previous sections. The general information about the photophysical properties of fluorescent markers used in live-cell research is gathered in Table 3. An interested reader can find more details about these fluorochromes in the cited literature.

**Table 3:** Generalized Optical Properties of Fluorescent Markers Used in Live-Cell (S)TEM Research for Cell Viability Testing

Fluorescent marker name	Specificity/properties	Excitation maximum, nm	Emission maximum, nm	Quantum yield in solution^a^	Ref
propidium iodide (PI)	nucleic acids membrane impermeable staining of dead cells	495	619	0.78	(195)
Syto 9	nucleic acids membrane permeable staining of living and dead cells	482	500	unbound to nucleic acids: <0.01; bound: >0.4	(196)
green fluorescent protein (GFP)^b^	functioning as a reporter gene gene expression evaluation staining of living cells	395	510	0.79	(194)
FUN-1	nucleic acids membrane permeable metabolic activity assessment staining of living cells	488	590	depends on the metabolic state of the cell	(181)
fluorescein diacetate (FDA)	no fundamental specificity membrane permeable nonfluorescent molecule fluorophore after hydrolysis staining of living cells	498	517	0.93	(197)

a PI – water; Syto 9 – not specified in the literature; GFP – buffer; fluorescein – 0.1 M NaOH.

b The optical properties vary depending on the specific type of GFP. All information can be found in the cited literature.

An issue connected to using fluorescent dyes after (S)TEM imaging is that the photobleaching process that could occur upon electron radiation is poorly described. GFP is the only fluorochrome studied as well in wet,^198^ as solid state,^199,200^ and the results showed that the optical properties change with increasing electron beam energies, including the emission peak shift and intensity changes in the cathodoluminescence spectra. Similar changes could appear for other fluorescent markers and thus lower the veracity of live-cell studies. Fluoresceine, a standard fluorescent dye, at a wavelength of 498 nm, can absorb up to about 2 × 10^4^ photons of energy equal to 2,54 eV each before the photobleaching process occurs.^201−203^ Thus, the cumulative energy of the absorbed photons is on the order of 4 × 10^4^ eV for a molecule. The dye concentration in a cell depends on its type and imaging technique and has the value of about 10^4^ molecules per cell in a standard imaging.^204^ In the case of an electron beam, at an accelerating voltage of 200 kV, each electron carries an energy of 200 keV. A cell treated like a 2D circle with a radius of 500 nm has a surface of about 8 × 10^5^ nm^2^; for dose conditions around 100 e^–^/nm^2^, it gives at least 8 × 10^7^ electrons per observable cell surface. The cumulative energy of electrons is then on the order of 8 × 10^7^ eV. Divided by the standard average dye concentration of 10^4^ molecules per cell, it gives 2 × 10^3^ eV per molecule. The energy transfer to a sample by an electron beam usually involves transferring only fractions of the original energy to the sample molecules. Even though this roughly estimated value is lower than for the photons needed for the photobleaching process of fluoresceine, this value is still high, and its quantity can change significantly with very slight electron dose changes. It should also be remembered that the dose and dose rate in TEM are generally measured per unit area (e^–^/nm^2^), and the interaction of the beam and the sample takes place in the sample volume, so for the same dose, the conditions of interaction can be different, for example for distinct sample thicknesses. It is worth mentioning, however, that there are methods for converting electron flux to units of absorbed radiation expressed in grays.^47,124^ However, additional processes, such as the radiolysis of fluorophore or generation of radicals and reactive oxygen species during imaging (described in Section 3), cannot be overlooked, making the photobleaching process in (S)TEM even more complicated.^205^ For this reason, a rigorous electron dose rate must be maintained during fluorescence-supported live-cell imaging. A fundamental study of the impact of the electron beam on optical and chemical properties of liquid fluorophores supported by spectroscopic methods (e.g., UV–vis, IR, Raman) is needed to avoid false interpretation of fluorescence cell viability tests after (S)TEM imaging.

To summarize this section, we could ask whether using techniques other than standard fluorescence imaging is possible. The answer is complex as it depends on the experiments. For precise (S)TEM studies of specific cells, fluorescence microscopy is probably the most reliable way to check whether the cells survived electron damage and could further reproduce. Flow cytometry offers a more precise quantitative analysis in general viability tests after (S)TEM imaging, where all cells were exposed to a damaging electron beam with a comparable dose. Still, in the case of a very small number of cells used in (S)TEM imaging (usually less than 50), its resolution might not be enough. Depending on cell size, the reliable number of cells in suspension that can be counted for flow cytometry within several seconds is up to 10000 for larger cells (yeast, animal cells) and up to 25000 for smaller cells (bacteria). Increasing the number of cells examined in such studies could enhance a more precise determination of the lethal dose.

## 6 Conclusions and Perspectives

A few conclusions can be drawn in light of the described experimental results and theoretical estimations. Various approaches were utilized for live-cell studies (Figure 6), including correlative light-(S)TEM imaging,^20^ electron holography,^26^ EELS analysis,^28^ and electron tomography.^29^ Each added valuable information to standard (S)TEM imaging. Subsequent studies could be enriched by using, for example, iDPC imaging.^134^ For most modern (after 2014) live-cell research, the electron dose was kept relatively low, with values ranging from 10 to 100 e^–^/nm^2^, and almost half of these works were supported by fluorescence microscopy imaging or cell growth studies (Table 2). However, the lethal electron dose for specific cell types is not clearly defined, and the “as low as possible” approach seems to be the most reasonable way to avoid significant cell damage. By analyzing Table 2, we could say that the highest electron dose used for imaging that did not kill the cells was 200 e^–^/nm^2^. Still, in this case, the fluorescence signal and the presence of a biomineralization process were the only methods that estimated the viability of the cells.^28^ Certain cells are more or less sensitive to radiation (as described in Section 4), and this also depends on the cell type, with vertebrate cells showing generally higher sensitivity while microbial cells (bacterial, yeast) lower.^206−209^ Furthermore, the microbial sensitivity to high-energy radiation is known to vary widely between different species of bacteria and even between different strains of the same species. It should also be noted that the intrinsic sensitivity of microorganisms and certain environmental factors, such as the presence of oxygen, water, or random water-soluble organic material, can also considerably affect the response of a given type of microorganism to radiation.^210^ Likewise, in animals, in the case of radioresistant tumor cells, the following factors can diminish their radiosensitivity: the enhanced ability to repair DNA damage, reduced oxygen access (hypoxic microenvironment), cell cycle position (cells are most resistant in G_0_, in early G_1_, and the late S phase of the cell cycle), and of course growth fraction.^211−213^ To our knowledge, the most radioresistant known organism is the Gram-positive bacteria *D. radiodurans*, accomplishing its resistance to radiation up to 10 kGy by having a unique cell wall, multiple copies of its genome, rapid DNA repair capacity, special structural features, and efficient antioxidant defense system.^144^ Many radio-resistant prokaryotic species can also be found among Archaea, including *Halobacterium*, *Pyrococcus*, or *Thermococcus* species that can withstand doses between 2.5 and 5 kGy without lethality.^214^ Therefore, a systematic study of the survival of all cell types on various substrates (SiN, graphene, carbon, and Formvar) under varying electron dose rates is needed to help determine the optimal imaging conditions. For such studies, fluorescence microscopy might not be enough to provide reliable results, as discussed in Section 5. The method of choice could be a relatively laborious task of counting cell reproduction since it could be challenging to implement for such a small number of microorganisms. In addition, these studies should focus on a higher number of cells tested in different areas of imaging (cells closer and further from the electron beam), as the general physiochemistry of irradiated cells in small volumes is still not well understood, and radiolysis products may diffuse through the whole sample.

For most of the research, silicon nitride was the substrate of choice for live-cell experiments. This type of liquid cell can precisely control the liquid thickness and offers liquid flow control in dedicated holders. However, thick SiN windows negatively impact cell visibility due to high electron scattering and may also lead to unfavorable charge accumulation. The use of graphene and its derivatives as radical scavengers may help obtain higher resolution and reduce the cell damage caused by electrons. However, sample preparation remains more complex than that for commercialized SiN-based liquid cells, and its repeatability is a concern. As shown in Table 2 and in Section 5, more GLCs have been implemented for live-cell research in recent years than in the past, including hybrid solutions by combining both SiN substrate and additional graphene coating. Both substrates supported cell culture growth, enabling biofilm formation growth and subsequent examination in (S)TEM. On the one hand, the thickness of SiN cells is relatively high, which makes (S)TEM observations difficult (especially when the electron dose needs to be low). Additionally, in certain experiments, the small volume of liquid cells might not be enough to observe specific processes occurring in living organisms, as mentioned before.^30^

As discussed earlier, Gram-positive and Gram-negative bacteria differ in peptidoglycan thickness, with the former having a thick peptidoglycan and the latter having a thin one. This feature is also crucial in LP-TEM imaging as hydrated Gram-positive bacteria give higher contrast than the Gram-negative ones.^34^ For this reason, it may be worth taking inspiration from the approach commonly used in cryoEM and considering the use of systems that utilize not only the amplitude-based but also the phase-based method of generating contrast based on the use of phase plates. Due to the entirely different sample thicknesses and scattering levels, this will probably require some change in the geometry of the phase plates. Another method of revealing the phase contrast may be using the already mentioned iDPC techniques.^134^ While aberration-corrected TEM has become a standard in high-resolution cryoEM and material science, it seems that for massive liquid cell samples, spherical aberration correction is an unnecessary setup complication. On the other hand, the use of energy filters for zero-loss peak image filtering is worth considering. High-sensitivity cameras should also complement the ideal microscope setup for live-cell TEM, so we can expect the use of direct detector cameras to increase in the coming years. The importance of the scavenging methods of radiolysis products is also likely to increase. Although this is a very advantageous method of working with soft matter samples, it should be remembered that scavenging of radiolysis products inside living organisms, if not impossible, remains an intriguing but future perspective.

This review highlights that live-cell research enables nanoscale observations in biological systems with a resolution that is not achievable by other microscopic techniques. Further advances in this approach could help to study yet not well-understood interactions of living cells with nanomaterials of different sizes, shapes, and composition,^215^ photosensitizers used for photodynamic therapy,^216^ drugs and nanodevices for nanomedicine,^217,218^ as well as biomineralization processes in species different than magnetotactic bacteria,^219−222^ and the influence of ionizing radiation or light.^223^ The correlative microscopic and spectroscopic methods should be used for the highest reliability of such studies. The fluorescence and confocal microscopy, as mentioned earlier, will still be crucial for basic imaging, but they could be extended to superresolution^6^ and nonlinear optical techniques^224^ with the use of novel dyes^225^ and nanomaterial-based fluorescent markers.^191,226,227^ It should also be kept in mind that any live-cell electron microscopy attempts will, in principle, be subject to strict evaluation and criticism because of the use of an imaging medium that, even at a minimal dose, potentially seriously damages the sample’s functions. Therefore, any experiments of this type should not be a replacement but rather a supplement to solidly established methods involving sample fixation, cryoEM methods, or less resolving light microscopy methods. It should also be noted that the absolute minimum of correctness when reporting experiments on live or even hydrated cells in electron microscopes is a very precise description of the electron dose. The next step may be survival tests but with a high degree of criticism and awareness of the potential limitations of the tests conducted. However, although extremely demanding, we do not believe that live cell TEM remains impossible.

## Vocabulary

fluorescent markers: substances that emit light upon excitation and utilized to label specific molecules or structures
in situ electron microscopy: real-time observations of material under external stimuli
liquid cell electron microscopy: technique of imaging liquid or hydrated samples using electron microscopy
live-cell electron microscopy: idea of imaging cells which remain viable during or after imaging
radical scavenger: chemical substance that absorbs and neutralizes radiolysis products yielding a protective function
radiolysis: chemical decomposition caused by ionizing radiation
viability: capability of demonstrating characteristics typical of life
